# Long-QT founder variant T309I-Kv7.1 with dominant negative pattern may predispose delayed afterdepolarizations under β-adrenergic stimulation

**DOI:** 10.1038/s41598-021-81670-1

**Published:** 2021-02-11

**Authors:** Iva Synková, Markéta Bébarová, Irena Andršová, Larisa Chmelikova, Olga Švecová, Jan Hošek, Michal Pásek, Pavel Vít, Iveta Valášková, Renata Gaillyová, Rostislav Navrátil, Tomáš Novotný

**Affiliations:** 1 Department of Medical Genetics, University Hospital Brno and Faculty of Medicine, Masaryk University, Jihlavská 20, 625 00 Brno, Czech Republic; 2 Department of Experimental Biology, Faculty of Science, Masaryk University, Kotlářská 267/2, 611 37 Brno, Czech Republic; 3 Department of Physiology, Faculty of Medicine, Masaryk University, Kamenice 5, 625 00 Brno, Czech Republic; 4 Department of Internal Medicine and Cardiology, University Hospital Brno and Faculty of Medicine, Masaryk University, Jihlavská 20, 625 00 Brno, Czech Republic; 5 Department of Biomedical Engineering, Faculty of Electrical Engineering and Communication, Brno University of Technology, Technická 10, 616 00 Brno, Czech Republic; 6 Division of Biologically Active Complexes and Molecular Magnets, Regional Centre of Advanced Technologies and Materials, Faculty of Science, Palacký University in Olomouc, Šlechtitelů 27, 783 71 Olomouc, Czech Republic; 7 Institute of Thermomechanics, Czech Academy of Sciences, Dolejškova 5, 182 00 Prague, Czech Republic; 8 Department of Paediatrics, University Hospital Brno and Faculty of Medicine, Masaryk University, Černopolní 9, 613 00 Brno, Czech Republic; 9 Repromeda, Clinic for Reproductive Medicine and Preimplantation Genetic Diagnosis, Biology Park, Studentská 812/6, 625 00 Brno, Czech Republic

**Keywords:** Biophysics, Computational biology and bioinformatics, Genetics, Molecular biology, Cardiology

## Abstract

The variant c.926C > T (p.T309I) in *KCNQ1* gene was identified in 10 putatively unrelated Czech families with long QT syndrome (LQTS). Mutation carriers (24 heterozygous individuals) were more symptomatic compared to their non-affected relatives (17 individuals). The carriers showed a mild LQTS phenotype including a longer QTc interval at rest (466 ± 24 ms vs. 418 ± 20 ms) and after exercise (508 ± 32 ms vs. 417 ± 24 ms), 4 syncopes and 2 aborted cardiac arrests. The same haplotype associated with the c.926C > T variant was identified in all probands. Using the whole cell patch clamp technique and confocal microscopy, a complete loss of channel function was revealed in the homozygous setting, caused by an impaired channel trafficking. Dominant negativity with preserved reactivity to β-adrenergic stimulation was apparent in the heterozygous setting. In simulations on a human ventricular cell model, the dysfunction resulted in delayed afterdepolarizations (DADs) and premature action potentials under β-adrenergic stimulation that could be prevented by a slight inhibition of calcium current. We conclude that the *KCNQ1* variant c.926C > T is the first identified LQTS-related founder mutation in Central Europe. The dominant negative channel dysfunction may lead to DADs under β-adrenergic stimulation. Inhibition of calcium current could be possible therapeutic strategy in LQTS1 patients refractory to β-blocker therapy.

## Introduction

Long QT syndrome (LQTS), the most often diagnosed familial electrical disease of the heart^1^, is characterised by a prolonged QTc interval and occurrence of polymorphic ventricular tachycardias of the *torsades de pointes* (TdP) type, degenerating into ventricular fibrillation in extreme cases. It results in syncopes or sudden cardiac deaths. Seventeen genes have been associated with LQTS so far^2,3^, while three "major" genes (*KCNQ1*, *KCNH2*, *SCN5A*) contribute to 75% of clinically diagnosed LQTS^4,5^. As confirmed recently, a substantial number of the other "minor" genes show only limited or disputed association with LQTS^6^.

Identification of the same mutation in apparently unrelated LQTS patients in a particular geographic region suggests derivation of the mutation from a common ancestor (a so-called “founder”). Several founder mutations in the *KCNQ1* gene associated with LQTS have been reported^7–19^. Founder populations have proven to be useful for studying genotype–phenotype correlations, which are important for patients´ management as prevalence of the disease can be increased in some geographical regions^20^.

This study is a complex analysis of the c.926C > T variant (p.Thr309Ile or T309I), the first founder mutation in the *KCNQ1* gene in Central Europe. Based on the acquired functional data, the underlying arrhythmogenic mechanism and possible alternative therapeutic way of its prevention were predicted by in silico modelling in a human ventricular cell model.

## Methods

### Clinical diagnostics

The study conformed to the principles outlined in the Declaration of Helsinki and was approved by the Multicenter Ethical Committee, University Hospital Brno (Brno, Czech Republic). All participants signed a written informed consent form. In the case of participants under the age of 18 years, the written informed consent was obtained from a parent and/or legal guardian.

LQTS diagnosis was established according to ESC Guidelines^21^. All individuals underwent clinical examination and bicycle ergometry. The initial stress was set to 0.5 W/kg, and increased by 0.5 W/kg every three minutes to achieve a heart rate higher than the submaximal value with respect to age and sex. A 12-lead ECG with Mason-Likar modification was employed. QT and RR intervals were measured manually (the end of QT interval was established using the threshold method); the Bazett’s correction formula was used. Detailed description is provided in the Supplementary Methods.

### Genetic analysis

Molecular analysis of LQTS-associated genes was performed according to current practises for molecular genetics diagnostics: multiplex PCR/SSCP analysis of 3 LQTS major genes (*KCNQ1, KCNH2* and *SCN5A*), Sanger sequencing on ABI 3100 Genetic Analyser (*Applied Biosystems*, Foster City, CA, USA; genes *KCNQ1, KCNH2* and *SCN5A*), and massive parallel sequencing MPS on GS Junior (*Roche*, Basel, Switzerland; genes *KCNQ1*, *KCNH2*, *SCN5A*, *KCNE1*, *KCNE2*) and, since 2016, on MiSeq (*Illumina*, San Diego, CA, USA; genes *KCNQ1*, *KCNH2*, *SCN5A*, *AKAP9*, *CACNA1C*, *CALM1*, *CALM2*, *CAV3*, *KCNE1*, *KCNE2*, *KCNJ5*, *SCN4B*, *SNTA1*). Genetic counselling and testing of first-degree relatives were offered to patients at risk.

The functional impact of the T309I variant was predicted with various in silico tools (SIFT, Provean^22^, MutationTaster, FATHMM^23^, and PMUT; Suppl. Tab. S1)^24^. Its conservation was measured with LRT and MutationAssessor. Allele frequency was determined from online databases ExAC^25^ and GnomAD.

For the haplotype analysis, 9 STR (short tandem repeats) markers spanning the ~ 11.9-Mb region of chromosome 11 (including the *KCNQ1* gene) were chosen from the UCSC Genome Browser (Suppl. Tab. S2). Multiplex PCR with fluorescently labelled primers and fragment analysis with capillary electrophoresis were performed on SeqStudio Genetic Analyzer (*Applied Biosystems,* Foster City, CA, USA). The haplotype linked to the mutation was identified by studying segregation in families. In the probands, SNP (single nucleotide polymorphism) marker analysis followed; 6219 SNPs on the p-arm of chromosome 11 were analysed. Population allele frequency analysis was performed after identifying a common STR allele in the marker D11S4088 in all mutation carriers. The control group was formed by 52 unrelated patients examined at the Department of Clinical Genetics, Faculty Hospital Brno, with a signed written informed consent from each patient agreeing that their DNA samples could be used for clinical research. STR alleles (104) were analysed with capillary electrophoresis. Detailed description is provided in the Supplementary Methods.

### Functional analysis

Plasmids containing wild-type (WT) human *KCNQ1* in a pIRES2-eGFP vector, *KCNE1* in a pKB-CMV vector, and *Yotiao* in a pGW1 vector were isolated from *Escherichia coli* using the endotoxin-free QIAprep Spin Miniprep Kit (*Qiagen*, Hilden, Germany). The mutation c.926C > T in the human *KCNQ1* (p.T309I) was generated by site-directed mutagenesis using QuikChange II XL Site-Directed Mutagenesis Kit (*Agilent Technologies*, Cedar Creek, TX, USA). Detailed description is provided in the Supplementary Methods.

TransFast Transfection Reagent (*Promega*, Madison, WI, USA) was used for transfection of the plasmids *KCNQ1*, *KCNE1* and *Yotiao* (the molar ratio 1:2:4, total amount of *KCNQ1* DNA 1 µg, ratio of DNA to transfection agent 1:1.5) into Chinese hamster ovary (CHO) cells cultured at 37 °C / 5% CO_2_ in the Ham’s F-12 medium supplemented with 10% foetal calf serum and 0.005% gentamycin. *KCNQ1* was transfected in one of three ways: 1) WT variant alone (1 µg; WT); 2) T309I variant alone (1 µg; T309I); 3) both the WT and T309I variants cotransfected in the ratio 1:1 (0.5 µg of WT *KCNQ1* and 0.5 µg of the T309I variant; WT/T309I) to mimic the heterozygous state in the mutation carriers.

Biophysical analysis was performed ~ 24 h after the transfection by the whole cell patch clamp technique at 37°C. The resistance of filled glass electrodes was < 2.5 MΩ the series resistance was compensated up to 60%. Tyrode solution of the following composition was used (in mmol/L): NaCl 132, KCl 4.8, CaCl_2_ 2.0, MgCl_2_ 1.2, HEPES 10, glucose 5 (pH 7.4, NaOH). The patch electrode filling solution contained (in mmol/L): K-aspartate 110, K_2_ATP 5, CaCl_2_ 1, MgCl_2_ 1, EGTA 11, HEPES 10 (pH 7.3, KOH). To simulate β-adrenergic stimulation, the pipette solution was supplemented with cyclic adenosine monophosphate (cAMP, 200 µmol/L) and okadaic acid (OA, 0.2 µmol/L). The junction potential was + 15 mV.

Considering missing proportionality between the measured current and estimated cell membrane capacitance, conversion of the magnitude of the current to the current density was avoided in this study, as recently recommended by Kula et al*.*^26^. The average cell membrane capacitance was comparable in cells expressing the wild type (WT), T309I, and WT/T309I *I*_Ks_ channels (13.8 ± 1.7, 13.3 ± 2.0, and 13.5 ± 1.8 pF, respectively; *P* > 0.05).

For the fluorescence image acquisition, a confocal laser scanning microscope Leica TCS SP8 X (*Leica microsystems*, Wetzlar, Germany) was used. WT and T309I human *KCNQ1* tagged with GFP at the 3′-terminus, or WT-*KCNQ1* without GFP in a pBK-CMV vector, as well as WT human *KCNE1* in a pBK-CMV vector were transfected into CHO cells seeded on glass bottom dishes (*Cellvis*, Mountain View, CA, USA) coated with fibronectin ~ 48 h before the evaluation (*KCNQ1* and *KCNE1* in the molar ratio 1:2). In some experiments, WT with no GFP was cotransfected with the GFP-tagged T309I-*KCNQ1* and *KCNE1*, and the cell membrane was stained (CellMask Orange Plasma Membrane Stain; *Invitrogen*, Carlsbad, CA, USA).

Detailed description is provided in the Supplementary Methods. The chemicals were purchased from *Sigma-Aldrich* (Prague, Czech Republic) unless otherwise indicated.

### Mathematical modelling

A previously published model of human ventricular subepicardial myocyte^27^ was modified and adapted to changes accompanying β-adrenergic stimulation induced by 1 µM isoproterenol (Suppl. Fig. S1, Suppl. Tab. S3; for validation of the new model, see Suppl. Fig. S3-S5). The measured gating properties of WT and WT/T309I *I*_Ks_ channels were incorporated into the model (Suppl. Fig. S6). Detailed description is provided in the Supplementary Methods. The Matlab code of the control model can be downloaded at https://www.it.cas.cz/en/d3/l033/.

### Statistical analysis

The data are mostly presented by the arithmetic mean (± SD from *n* patients, or ± SEM from *n* cells; Origin, version 8.5.1; *OriginLab Corporation*). To determine the statistical significance of the differences, paired/unpaired *t*-tests and one-way/repeated measures ANOVA with the Bonferroni post-test were performed using the software GraphPad Prism, version 6.05 (*GraphPad Software, Inc.*). The same software was used for curve fitting. If the difference between the arithmetic and geometric means was > 10% (considering a recent study by Kula et al*.*^28^), the geometric mean x/ geometric SE and the non-parametric Mann–Whitney test were used (Figs. 2B, 3B). *P* < 0.05 was considered statistically significant.

To compare the statistical significance of differences in relative fluorescence intensity in Fig. 4 (where several data samples did not show the normal distribution according to the Shapiro–Wilk test; the mean values are represented by the geometric mean ± 95% confidence interval), either the Friedman test or the Kruskal–Wallis test (both with the Dunn´s multiple comparison) were used, the first one in the case of paired data (comparison within individual groups), the latter in the case of unpaired data (comparison among various groups).

**Figure 1:**
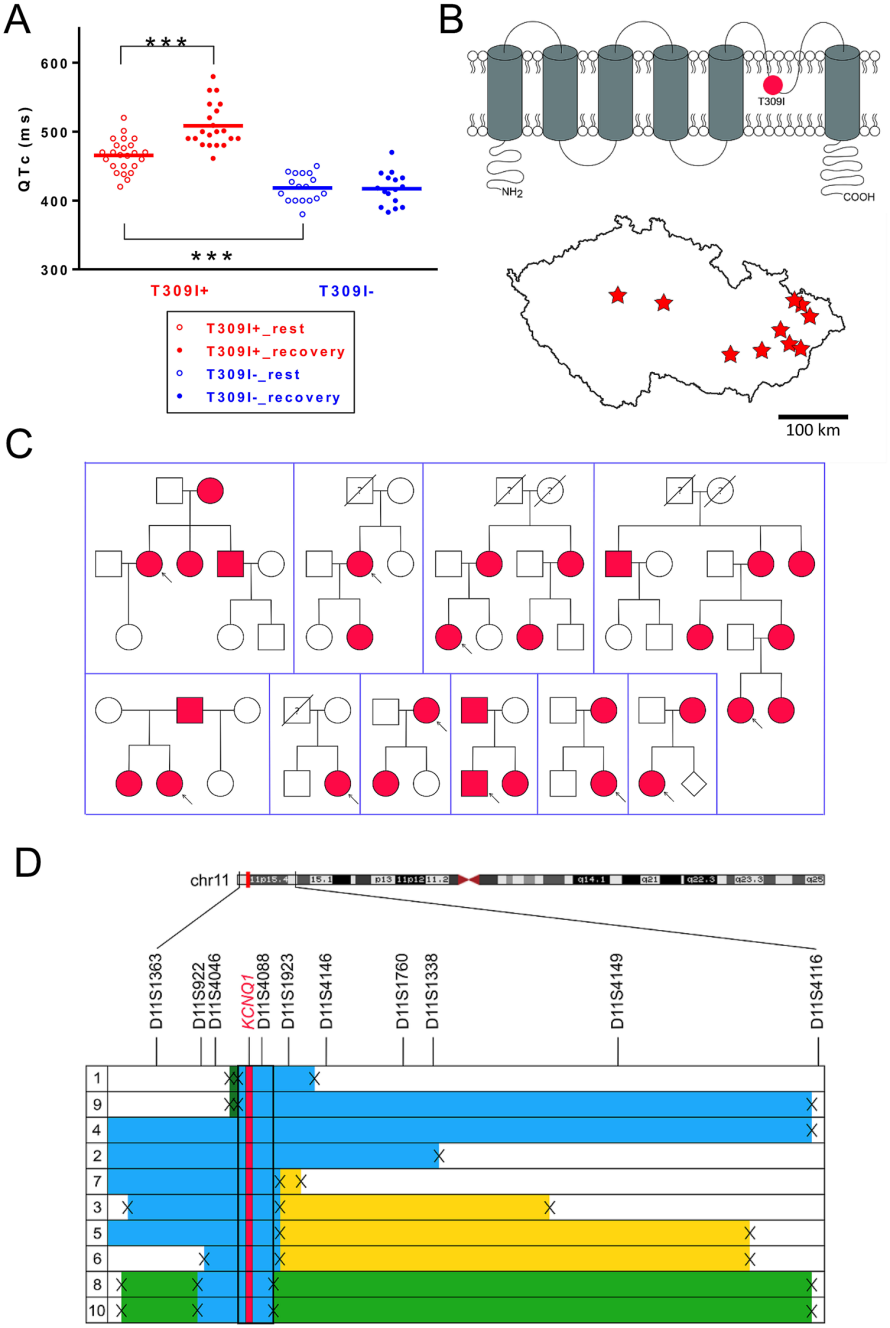
Clinical and genetic characterization of the T309I variant. (**A**) QTc interval length in the heterozygous T309I carriers (T309I +) and their unaffected relatives (T309I-), both at rest and in the 4th minute of exercise test recovery; ***statistical significance at *P* < 0.001; the graph was prepared using the software GraphPad Prism, version 6.05. (**B**) Scheme of T309I-Kv7.1 subunit (upper panel) and birthplaces of the oldest T309I carriers in every family (lower panel; the map was generated using the software QGIS, version 3.10, with data downloaded from https://www.arcdata.cz/produkty/geograficka-data/arccr-500). In the 2 families located in the central part of the country, some earlier ancestors from the eastern part could be identified. Nevertheless, none of them has been living at the time of the study and biological material was not available for molecular analysis. Thus, we can only speculate, whether these ancestors coming from the east really carried the variant, even if it is truly anticipated. (**C**) Family pedigrees; affected individuals in red, probands indicated with arrows. (**D**) Haplotype analysis of the *KCNQ1* gene and surrounding regions (analysed STR markers shown in relative distances). The region shared by all affected individuals is framed by bold lines. Families with the same detected haplotype are divided into 3 subgroups (differentiated by colours); presumable ancestral haplotype is shown in blue. The uncoloured parts are unique in every single family; small crosses mark where crossing-overs occurred. Parts B, C, and D were prepared using the software Inkscape, version 0.92.2.

**Figure 2:**
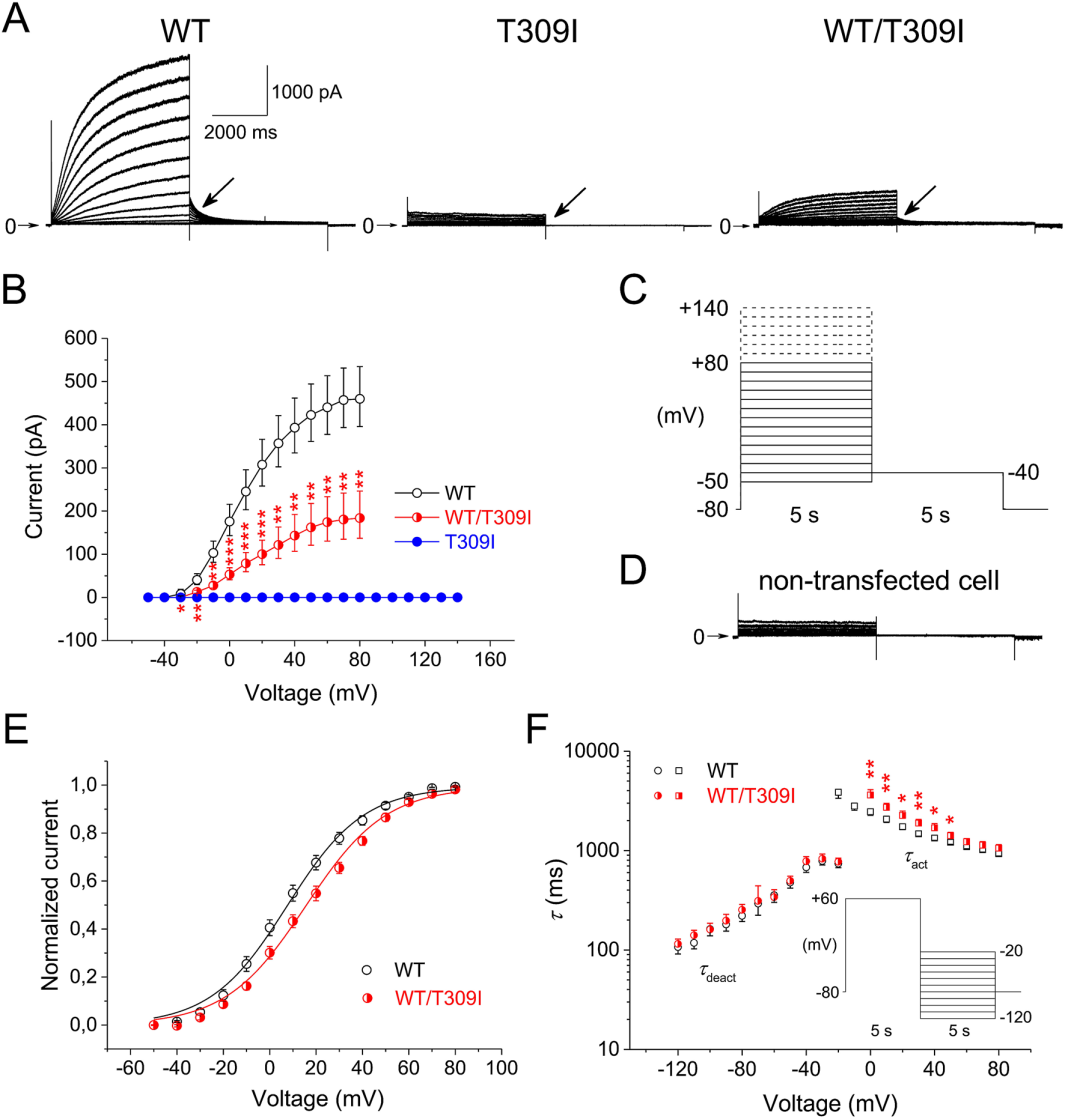
Biophysical impact of T309I mutation. (**A**) Representative records in wild type (WT), T309I, and WT/T309I channels (KCNE1 subunit cotransfected); arrows point to the tail current. (**B**) Average voltage dependence of tail current activation (*n* = 20, 9, and 16 in WT, T309I, and WT/T309I channels, respectively); the half-maximal activation voltage *V*_1/2_ = 8.3 ± 2.2 mV in WT channels and 15.6 ± 2.1 mV in WT/T309I channels; *P* < 0.05). (**C**) Activation experimental protocol; the dashed lines—steps used for stimulation only in T309I channels. (**D**) Representative trace from a non-transfected CHO cell. (**E**) Average voltage dependence of steady-state activation. (**F**) Average voltage dependence of the time constant of activation (τ_act_) and time constant of deactivation (τ_deact_; *n* = 11 and 9 in WT and WT/T309I channels, respectively); inset: deactivation experimental protocol; *, ** and ***statistical significance between WT and WT/T309I channels at *P* < 0.05, 0.01 and 0.001, respectively. The stimulation frequency was 0.08 Hz. All graphs were prepared using the software Origin, version 8.5.1.

**Figure 3:**
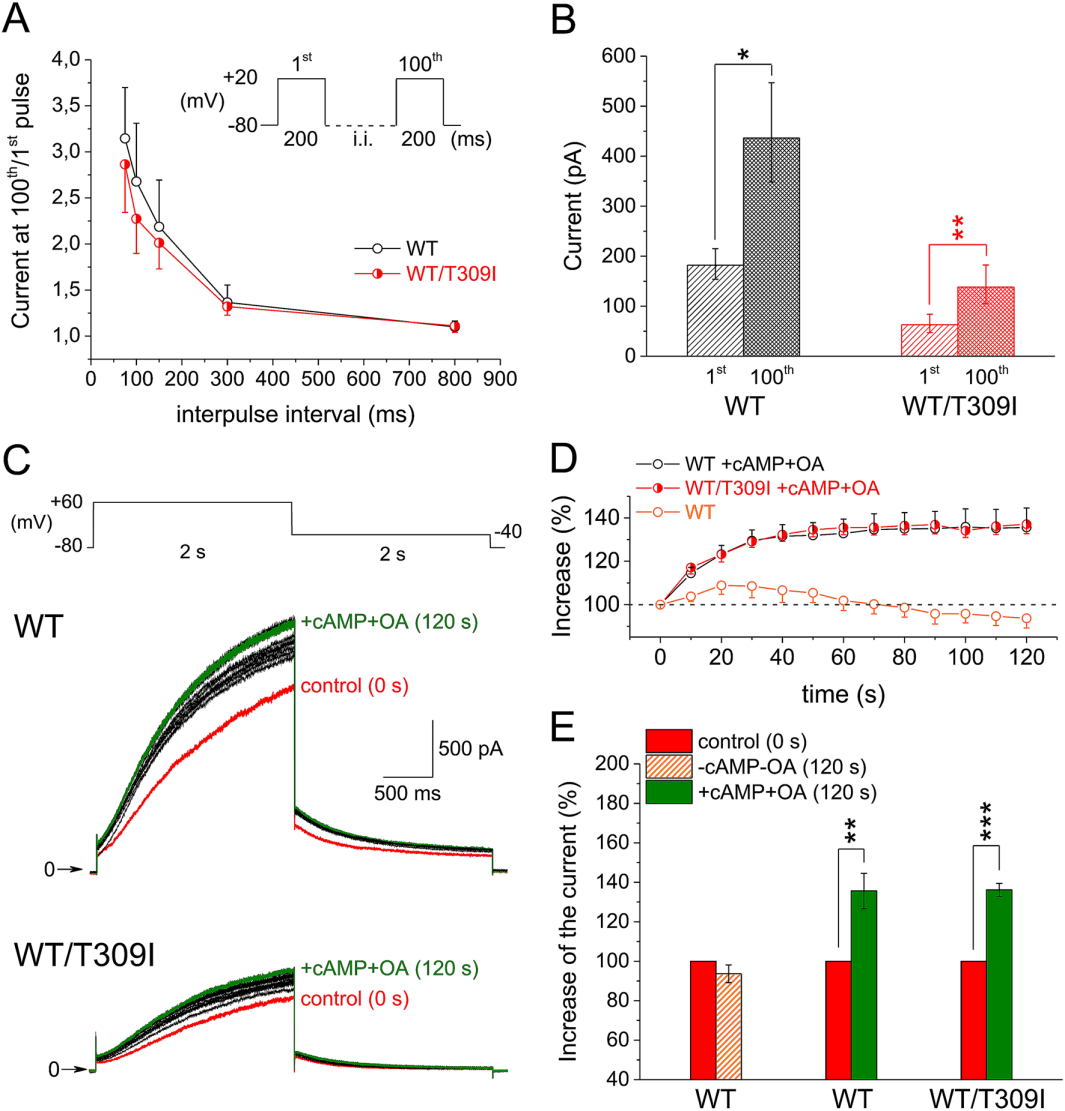
Accumulation of WT and WT/T309I currents (KCNE1 subunit cotransfected). (**A**) Average relative rate-dependent accumulation of the currents at interpulse intervals between 75 and 800 ms (*n* = 10 and 8 in WT and WT/T309I channels, respectively); inset: experimental protocol applied after a 30-s pause; i.i.—interpulse interval. (**B**) Average absolute currents at the 1st and 100th pulse of the accumulation protocol with the interpulse interval of 100 ms. (**C**) Experimental protocol (the stimulation frequency 0.1 Hz), and representative traces of WT and WT/T309I currents under simulated β-adrenergic stimulation (cAMP and OA in the pipette solution); red trace—time 0, control (no cAMP/OA stimulation); green trace—time 120 s, steady-state β-adrenergic stimulation; black traces—time 10 to 110 s in 10-s intervals, development of β-adrenergic response. (**D**) Time course of the relative increase of the current in presence of cAMP and OA (+ cAMP + OA) in WT and WT/T309I channels (*n* = 14 and 8; *n* = 9 in WT without cAMP and OA). (**E**) Relative steady-state increase of the current in + cAMP + OA; *, ** and ***statistical significance at *P* < 0.05, 0.01 and 0.001, respectively. All graphs were prepared using the software Origin, version 8.5.1.

**Figure 4:**
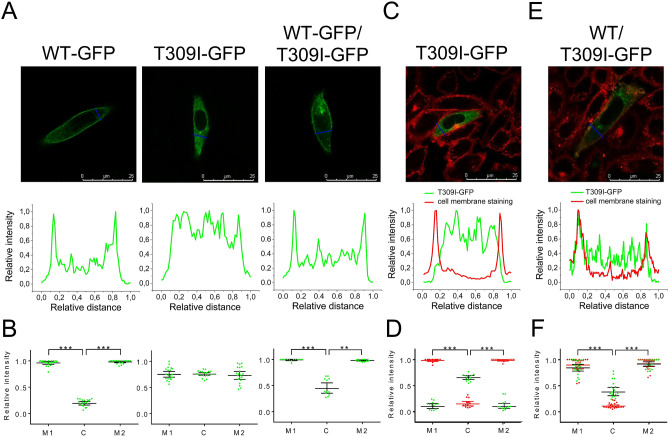
Subcellular localization of WT- and T309I-Kv7.1 subunits expressed in CHO cells (KCNE1 subunit cotransfected). (**A**) Confocal microscopic images of representative cells expressing WT-GFP (*i.e.* tagged with GFP at the C-terminal end), T309I-GFP, or both WT-GFP and T309I-GFP subunits (upper panels), and respective relative fluorescence intensity profiles (lower panels) under the blue lines in the upper panels; scale bar 25 µm. (**B**) The resulting maximal relative fluorescence intensity at the cell membrane (M1 and M2) and its mean value in the cytosol (**C**) in all investigated cells (*n* = 20, 20 and 12 in WT-GFP, T309I-GFP and WT-GFP/T309I-GFP subunits, respectively; cells from 4 to 7 transfections used in each variant); ** and ***statistical significance of the difference in fluorescence intensity among M1, M2 and C in the respective group of cells at *P* < 0.01 and 0.001, respectively. (**C**,**E**) Subcellular localization of the T309I-GFP subunits, either expressed alone (**C**) or co-expressed with non-tagged WT (no GFP; E); the cell membrane was stained with red fluorescent dye. Representative cells (upper panels) and respective relative fluorescence intensity profiles (lower panels) under the blue lines in the upper panels of the GFP signal (green lines) and of the membrane staining signal (red lines; both at the cell cross-sections indicated by the blue lines in the upper panels). (**D**, **F**) The resulting maximal relative fluorescence intensities of both GFP and membrane staining fluorescence (green and red dots, respectively) at the cell membrane (M1 and M2) and their mean values in the cytosol (C) in all investigated cells (*n* = 13 and 20 in D and F, respectively; cells from 3 and 7 transfections used); ***statistical significance of the difference in fluorescence intensity among M1, M2 and C at *P* < 0.001. Figure 4A,C, E were prepared using the software LAS X, version 3.5.2.18963 (upper panels) and Origin, version 8.5.1 (lower panels), Fig. 4B,D,F using the software GraphPad Prism, version 6.05.

Following software was used to prepare the figures: Figs. 1A and 4B,D,F—GraphPad Prism, version 6.05 (*GraphPad Software, Inc.*); Fig. 1B,C,D—Inkscape, version 0.92.2 (*Inkscape Project*; https://inkscape.org/cs/about/branding/; the map in Fig. 1B, lower panel, was generated using the software QGIS, version 3.10, with data downloaded from https://www.arcdata.cz/produkty/geograficka-data/arccr-500); Figs. 2, 3 and 4A,C,E (lower panels, and 5—Origin, version 8.5.1; *OriginLab Corporation*); Fig. 4A,C,E (upper panels)—LAS X, version 3.5.2.18963 (*Leica Microsystems CMS GmbH*).

## Results

### Clinical characteristics of T309I carriers

Members of 10 putatively unrelated families with occurrence of the T309I variant were investigated (Table 1). All of the 4 syncopes and 2 aborted cardiac arrests (ACA) which were reported in the investigated T309I carriers appeared during physical exertion (ice hockey, swimming, exercise at school). QTc intervals were significantly longer in T309I carriers compared to their healthy relatives, both at rest (466 ± 24 ms vs. 418 ± 20 ms, *P* < 0.001) and in the fourth minute of exercise test recovery (508 ± 32 ms vs. 417 ± 24 ms, *P* < 0.001; Fig. 1A). The QTc prolongation after exercise was significant in T309I carriers (*P* < 0.001). Representative ECG traces at rest and during recovery in symptomatic and asymptomatic T309I carriers are shown in Suppl. Fig. S7. In children under 16 years, the available QTc values were similar to the values in the adult population of the respective patient group (Table 1). No significant changes were apparent in PQ interval and QRS complex (Suppl. Fig. S8). Cardioverter defibrillators were implanted in the 2 ACA cases. In the other T309I carriers, β-blocker therapy (preferably with nadolol) has been administered and no syncope recurrences have been observed since.

**Table 1:** Clinical characteristics of investigated individuals, members of 10 unrelated families.

All individuals (n = 54)		
	T309I + (*n* = 30)	T309I- (*n* = 24)
Age (years)	30.0 ± 20.2	32.2 ± 20.1
Females	25 (83.3%)	13 (54.2%)

Individuals with completed clinical investigation (n = 41)		
	T309I + (*n* = 24)	T309I- (*n* = 17)
Age (years)	26.3 ± 18.4	30.1 ± 15.6
Females	20 (83.3%)	9 (52.9%)
Children under 16 years	10	3
Syncope/ACA	4/2	0/0
QTc, rest (ms)	466 ± 24^†††^	418 ± 20
QTc, rest—children (ms)	465 ± 27^††^	410 ± 10
QTc, rest—adults (ms)	466 ± 22^†††^	420 ± 21
QTc, recovery (ms)	508 ± 32***^†††^	417 ± 24
QTc, recovery—children (ms)	510 ± 40**^†^	425 ± 7
QTc, recovery—adults (ms)	508 ± 27***^†††^	416 ± 25

T309I + , T309I carriers; T309I-, T309I non-carriers; mean ± SD, *n*—number of individuals; QTc, QT interval corrected to the heart rate (Bazett´s formula); QTc, recovery, QTc, 4th minute of the recovery phase after ergometry; ACA, aborted cardiac arrest; statistics in the respective groups of patients (all/children/adults): ** and ***—statistical significance of QTc difference at rest and during recovery at *P* < 0.01 and 0.001, respectively, ^†^, ^††^ and ^†††^—statistical significance of the respective QTc difference between T309I + and T309I- at *P* < 0.05, 0.01 and 0.001, respectively.

### Genetic characteristics of families with T309I variant

The same substitution c.926C > T (NM_000218.2) in the *KCNQ1* gene (Suppl. Fig. S9) resulting in T309I amino acid change in the P-loop of Kv7.1 protein (Fig. 1B, upper panel) was identified in 10 unrelated probands, and in 30 carriers in total (Fig. 1C; heterozygous carriers in all cases). The birthplaces of the oldest known T309I carrier in every family are shown in Fig. 1B, lower panel.

The in silico predictive tools which were used suggested a damaging or disease-causing effect of T309I variant and evolutionary analysis showed high conservation of threonine at this position across species (Suppl. Fig. S9). The T309I variant was found neither in the ExAC nor in the GnomAD online databases.

The same haplotype was identified by STR analysis in the region nearest to the mutation spot in all T309I carriers (Fig. 1D). The same allele was detected in 100 SNPs surrounding the variant in all probands with SNP analysis, which also delimited the maximal size of the area shared by all affected individuals to 658,407 bp. This area contained the whole *KCNQ1* gene.

STR and SNP analysis of markers lying more distant from the *KCNQ1* gene identified 3 subgroups of families sharing the same haplotype spanning longer chromosome regions (Fig. 1D). SNP analysis also identified possible crossing-over spots (crosses in Fig. 1D). The maximum size of the area shared by two families (8 and 10) was delimited with 1847 SNPs and measured 12,633,501 bp. Genealogical analysis also revealed that the oldest carriers of T309I variant in these two families came from two neighbouring villages.

In the marker D11S4088 (the closest one to the *KCNQ1* gene), the same fragment with a length of 211 bp segregating with T309I variant was detected in all families. This marker was highly polymorphic in a control population; the fragment lengths varied between 203 and 253 bp. The 211-bp fragment was detected in 13 out of 104 chromosomes, resulting in allele frequency of 0.125. The probability that 10 unrelated people would have this allele is negligible (9.5 × 10^–7^).

### Impact of T309I variant on I_Ks_ channel function

All functional data were measured in the presence of KCNE1 subunits. If not otherwise stated, the stimulation frequency was 0.08 Hz. Considering the study by Kula et al*.*^26^, conversion of the magnitude of the measured current to the current density was avoided (for details, see Methods).

The homozygous T309I channels resulted in a complete absence of *I*_Ks_ (Fig. 2A,B), even if stimulated up to + 140 mV (for the experimental protocol, see Fig. 2C). The membrane current responses in non-transfected CHO cells did not differ from those in homozygous T309I channels (Fig. 2D).

In the heterozygous WT/T309I channels, *I*_Ks_ amplitude was significantly decreased compared to WT channels (Fig. 2A,B). The dysfunction showed characteristics of dominant negativity—WT/T309I current was supressed by 82 to 55% at voltages between -30 and + 20 mV (*i.e.* at voltages relevant for the cardiac action potential). The voltage dependence of steady-state activation was significantly shifted to the right in WT/T309I channels (Fig. 2E; the half-maximal activation voltage *V*_1/2_ = 8.3 ± 2.2 mV in WT channels and 15.6 ± 2.1 mV in WT/T309I channels, *P* < 0.05); the slope factors *k* remained unaltered (15.7 ± 0.7 and 17.3 ± 0.8). The time course of activation was significantly decelerated in WT/T309I channels at voltages between 0 and + 50 mV (Fig. 2F; the time constant of activation τ_act_ at + 20 mV: 1740 ± 110 ms in WT channels and 2254 ± 240 ms in WT/T309I channels, *P* < 0.05). No significant changes of the time constant of deactivation (τ_deact_) were observed (Fig. 2F; 220.5 ± 26.9 ms in WT channels and 253.6 ± 33.8 ms in WT/T309I channels at − 80 mV; the experimental protocol shown in the inset of Fig. 2F).

Subsequently, sets of hundred 200-ms pulses from -80 to + 20 mV were applied after a 30-s pause (inset in Fig. 3A). *I*_Ks_ amplitude was not significantly different during the 1st and 100th pulse during stimulation with the interpulse interval of 800 ms (Fig. 3A). At shorter interpulse intervals, *I*_Ks_ accumulated in both WT and WT/T309I channels, up to a similar extent in the relative scale (Fig. 3A; for data in absolute values at the interpulse interval of 100 ms, see Fig. 3B). Accumulation of *I*_Ks_ during simulated β-adrenergic stimulation (cAMP and OA in the pipette solution) was preserved in WT/T309I channels, being similar in WT and WT/T309I channels in the relative scale (reaching relative steady-state increase of the tail current by 36.2% in WT/T309I channels on average, *vs.* 35.6% in WT channels; Fig. 3C–E). As demonstrated in Fig. 3B,C, the preserved reactivity of WT/T309I channels to both the high-rate and β-adrenergic stimulations was not sufficient to reach adequate *I*_Ks_ magnitude (not reaching even the level of the WT current before start of the stimulations).

### Subcellular localization of I_Ks_channels with T309I variant

Since *I*_Ks_ was absent in the homozygous T309I channels (Fig. 2A,B), the subcellular localization of WT and T309I subunits (in the presence of KCNE1) was studied. WT-GFP channels showed clear fluorescence preferentially localized at the cell membrane (Fig. 4A,B, left panels; *P* < 0.001). In contrast, T309I-GFP channels seemed to be retained inside of the cell (Fig. 4A,B, middle panels). To prove this hypothesis, experiments with cells expressing T309I-GFP channels were repeated in a new set of cells, with the cell membrane marked by a red fluorescent dye. GFP fluorescence tagged with T309I subunits (green line/dots) was detected only inside of the cells, *i.e.* not in the cell membrane (red line/dots; Fig. 4C,D).

When WT-GFP and T309I-GFP subunits were co-transfected, the GFP fluorescence signal was present both on the cell membrane and inside the cells (Fig. 4A,B, right panels). It was significantly more intense on the cell membrane (*P* < 0.001). The relative intensity of the fluorescence signal at cytosol was significantly different in all tested transfection variants (Fig. 4B; *P* < 0.001 in WT vs. T309I, *P* < 0.01 in WT/T309I vs. T309I, and *P* < 0.05 in WT vs. WT/T309I). This suggests that trafficking is impaired in the WT/T309I channels; however, contribution of T309I subunits to formation of the cell membrane channels is likely. To prove this hypothesis, channels composed of WT subunits without GFP and T309I subunits tagged with GFP (WT/T309I-GFP) were prepared, and the cell membrane was marked by red fluorescent dye. The fluorescence intensity profile peaks of the cell membrane staining (red line/dots) and of the GFP fluorescence tagged with T309I subunits (green line/dots) overlapped (Fig. 4E,F). This implies that T309I subunits contributed to the formation of *I*_Ks_ channels localized in the cell membrane when co-expressed with the WT subunits.

### Functional impact of T309I variant in human cardiac cell model

**Figure 5:**
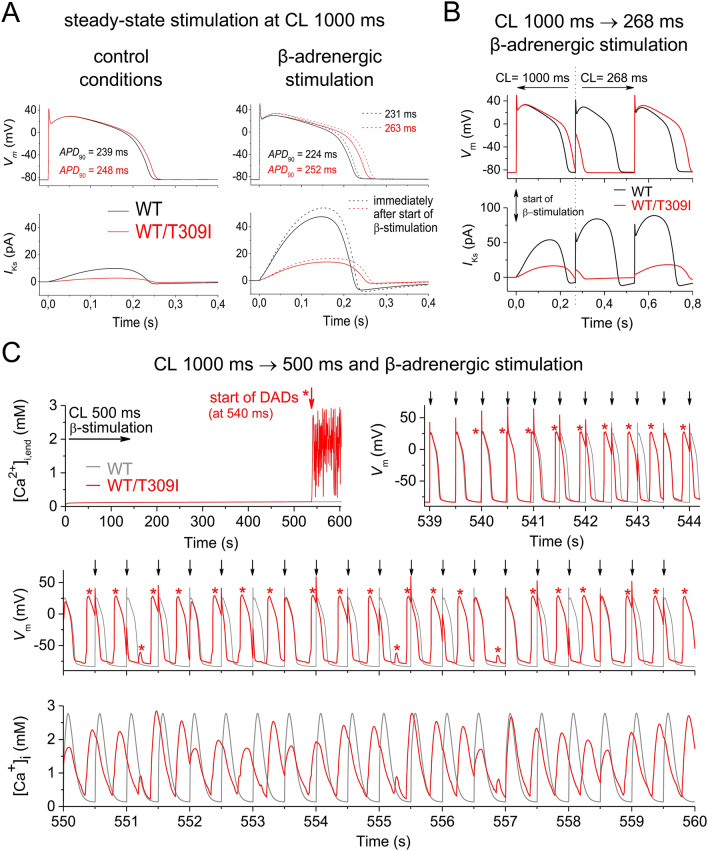
Impact of WT/T309I dysfunctional channels on action potential (AP) configuration in a human ventricular cell model. (**A**) APs (upper panels) and *I*_Ks_ (lower panels) at steady-state stimulation at the cycle length (CL) 1000 ms in control conditions and during β-adrenergic stimulation; *APD*_90_—AP duration at 90%-repolarization. (**B**) APs (upper panels) and *I*_Ks_ (lower panels) at extrastimulation within the vulnerable period of AP in the WT/T309I model during β-adrenergic stimulation—CL suddenly shortened from 1000 to 268 ms. (**C**) Intracellular Ca^2+^ concentration at the end of stimulation cycle ([Ca^2+^]_i,end_; left upper panel), APs (right upper and middle panels) and intracellular Ca^2+^ transients ([Ca^2+^]_i_; lower panel) after change of CL from 1000 to 500 ms and contemporary start of β-adrenergic stimulation (for sequence of changes, note the time axes in all graphs); arrows—stimuli at CL 500 ms, *delayed afterdepolarization (DAD) or AP activated by DAD. All graphs were prepared using the software Origin, version 8.5.1.

At slow steady-state stimulation (cycle length, CL, 1000 ms), the action potential duration at 90%-repolarization (*APD*_90_) was only slightly prolonged in the model with WT/T309I channels in control conditions (by 3.8% vs. the WT model; Fig. 5A, left panel). This agrees with the well-known limited impact of *I*_Ks_ on action potential configuration at low stimulation rates^29–31^. Under β-adrenergic stimulation (Fig. 5A, right panel), *APD*_90_ decreased by 6.3% in the WT model (as usual^32^) but increased by 1.6% in the WT/T309I model. This resulted in substantially longer *APD*_90_ in the WT/T309I vs. WT model at β-adrenergic stimulation (by 12.5%, or, during the first action potential after start of β-adrenergic stimulation, even by 13.9%).

If extrastimulation was applied within the vulnerable period of the first WT/T309I action potential after the start of β-adrenergic stimulation, the first action potential was suppressed and the following one was extremely prolonged in the WT/T309I model (it was longer by 31% in comparison with WT model; Fig. 5B). Irregular suppression of action potentials and action potential alternans followed (not illustrated). To provoke this, CL had to be decreased below 269 ms in the WT/T309I model, and below 238 ms in the WT model.

If CL was shortened to 500 ms and β-adrenergic stimulation was started at the same time (*i.e.* conditions comparable to the start of physical activity), the prolonged action potential in the WT/T309I model under β-adrenergic stimulation resulted in Ca^2+^ overload and development of delayed afterdepolarizations (DADs), often eliciting a premature action potential, within 540 s (Fig. 5C).

## Discussion

In this study, we performed a detailed description of the c.926C > T-*KCNQ1* (p.T309I-Kv7.1) variant, the first identified founder LQTS mutation in Central Europe. The dominant negative dysfunction of T309I *I*_Ks_ channels was observed in the heterozygous setting. Mathematical modelling demonstrated formation of DADs and action potential alternans at short CLs during β-adrenergic stimulation.

### Founder effect of T309I variant

The T309I variant was identified almost simultaneously in a Taiwanese family^33^ and in our earlier report^34^. No functional data has been available so far.

As the T309I variant was detected in 10 probands in our cohort of 44 LQTS1 families, its prevalence is unusually high in our region^25^. Majority of these 10 carrier families is geographically clustered (Fig. 1B, lower panel). Such characteristics are typical for mutations derived from the founder effect. Using detailed haplotype analysis of both STR and SNP markers, we confirmed that all affected individuals shared the same haplotype spanning ~ 658 kbp including the complete coding region of the *KCNQ1* gene (Fig. 1D). Allelic variability at a greater distance from the mutation spot can be explained by crossing overs in T309I ancestors. We identified three subgroups of haplotypes among the 10 investigated families, which are shared also in distant markers. The maximum length of the shared haplotype between two families (8 and 10) was ~ 12.63 Mb, and was represented by the same allele in the 1847 SNPs analysed (Fig. 1D). Hence, these two families are more closely related, which also correlates with their geographical origin. A close common ancestor could be also predicted in two other subgroups of families with shared haplotypes.

### Functional defect in T309I I_Ks_ channels

Considering the position of the T309I-Kv7.1 variant in a highly conserved pore helix^35^ and the replacement of the small polar residue by the bigger non-polar one, serious functional defect may be expected.

*I*_Ks_ was completely missing in the homozygous T309I channels (Fig. 2A,B) due to impaired channel trafficking (Fig. 4A,B), similarly as in the near variant A302V^36^ and in some other pore-region LQTS1 variants^37,38^. However, other *I*_Ks_ pore region variants showed unaffected trafficking^39,40^. This implies that only specific residues within the *I*_Ks_ pore play a role in the process.

In the heterozygous WT/T309I channels, dominant negativity was observed (Fig. 2A,B), as is often found in the pore-region LQTS1 variants^36–42^. A rightward shift of the voltage dependence of channel activation (Fig. 2E), which was also detected in some other pore-region LQTS1 variants^37,38,40–42^, suggests that even pore residues can modify the voltage sensitivity of the *I*_Ks_ channel. It has to be noted that variable expression ratio of *KCNQ1* and *KCNE1* in WT and WT/T309I channel complexes might contribute to the rightward activation shift. In WT/T309I channels, we also observed significantly decelerated activation kinetics (Fig. 2F). No other study analysing functional defects in the pore-region LQTS1 variants that we have found reported data on the activation kinetics; thus, we cannot assume that these variants generally exert an analogical effect. Response to β-adrenergic stimulation was preserved in WT/T309I channels (Fig. 3C–E), comparable to that reported by Spätjens et al.^31^ under similar conditions. Since the P-loop mutations studied so far showed unaltered β-adrenergic *I*_Ks_ accumulation^43^, P-loop is not likely involved in this essential regulation of *I*_Ks_ channel function.

Considering the data presented in Fig. 4E,F, a reverse way of cotransfection of WT and T309I subunits (WT tagged with GFP and T309I without GFP) might help to elucidate if a trafficking defect of WT subunits contributes to the observed *I*_Ks_ channel dysfunction described in Fig. 2. Comparing the intracellular GFP signals in Fig. 4B (right panel) and 4F (being not significantly different, *P* > 0.05), it seems that most of the subunits retained inside the cell are T309I subunits if both WT and T309I subunits are coexpressed. However, *I*_Ks_ channel is a heterotetrameric channel that is indeed formed by various combinations of WT and T309I subunits if both are transfected (and if even the mutated T309I subunit is present on the cell membrane, Fig. 4E,F). Taking all this into account, we speculate that a slight intracellular retention of WT subunits might happen if WT and T309I subunits are coexpressed.

### Genotype—phenotype correlation in the T309I variant

A mild LQTS phenotype was observed in the heterozygous T309I carriers, similarly to many other founder variants^14,44^ and also to some other pore region LQTS1 variants^36,40,42^. Surprisingly, a dominant negative dysfunction was revealed in the heterozygous T309I channels (Figs. 2 and 3). A similar contradiction between the channel dysfunction and clinical phenotype was described in the near A302V variant^36^. The mild T309I phenotype likely results from the preserved reactivity of the heterozygous mutated channels to high-rate and β-adrenergic stimulations (Fig. 3). This may partially compensate the dominant negative dysfunction at situations when *I*_Ks_ is essential for proper cardiac cell repolarization, namely during exercise or stress.

As predicted by in silico modelling on a human ventricular cell model, stimulation with shortened CL during β-adrenergic stimulation may trigger proarrhythmic activity in patients with WT/T309I *I*_Ks_ channels (Fig. 5B,C). DADs were observed, which even promoted premature action potentials. This observation may seem unexpected considering previously published studies which typically demonstrated occurrence of EADs in LQTS1 under β-adrenergic stimulation, including studies performed on patient-specific derived cardiomyocytes^45–47^ and on animal models^48–50^. According to other studies, EADs as an arrhythmogenic mechanism seem to be preferentially connected to LQTS type 2 which is based on mutations in the *KCNH2* gene (the rapid delayed rectifier current *I*_Kr_ is affected) and in which the TdP arrhythmia is usually pause-dependent^51,52^. In contrast, pause-independent TdP has been observed in LQTS1^52^. The fast heart rate preceding TdP (absence of pause) in LQTS1 is compatible with the mechanism of genesis of DADs^53^. Genesis of DADs at fast rates during β-adrenergic stimulation accompanied by *I*_Ks_ dysfunction (both pharmacologically-induced) was demonstrated in canine cardiac cells and tissue^54,55^, similarly as we have seen it in our human ventricular cell model with WT/T309I channels (Fig. 5C). The above listed contradictory data on LQTS1 arrhythmogenic mechanism suggest necessity of a further detailed study within this field.

In our model, the development of DADs was caused by an increase of sodium-calcium exchange current and calcium-activated non-specific current, due to a premature release of Ca^2+^ from the sarcoplasmic reticulum at intracellular Ca^2+^ overload, which in turn was caused by prolonged action potentials at the fast heart rate (for details, see Suppl. Fig. S10). This proarrhythmic events predicted by in silico modelling may explain the occurrence of arrhythmias during physical exertion in T309I carriers (see “Clinical characteristics of T309 I carriers” ).

The essential proarrhythmogenic role of Ca^2+^ overload suggested by in silico modelling should be considered in clinical practise in the future. It implies that, beside the standard treatment with β-blocking agents, additional or modified treatment may be beneficial in some LQTS1 patients, namely those who insufficiently respond to the standard β-blocking therapy. As demonstrated in Suppl. Fig. S11, a slight (5%) inhibition of the cardiac calcium current *I*_Ca_ is able to prevent development of DADs in our WT/T309I model during the tested period of 600 ms with a negligible effect on the magnitude of the intracellular Ca^2+^ transient (thus, likely not significantly impairing cardiac contractility). It implies that possible new treatment strategy of low doses of verapamil might be relevant in some LQTS1 patients, similarly as in patients with refractory catecholaminergic polymorphic ventricular tachycardia^56^. In patients with compromised cardiac contractility (in whom even a mild *I*_Ca_ inhibition would be contraindicated), a decrease of the late sodium current (*I*_Na,late_) might be beneficial^57,58^.

### Limitations of the study

Use of the human ionic channels heterogeneously expressed in a cell line limits possibilities of direct experimental investigation of impact of a particular mutation on complex cardiac cell electrophysiology. Therefore, it might be beneficial to prove the data in another model, for example in isolated cardiomyocytes of the affected patient/s (which is however very problematic, if not impossible, from ethical reasons), or in patient-specific cardiomyocytes derived from the induced pluripotent stem cells. Likewise, such more complex in vitro models might be helpful to better validate the human ventricular cell in silico model. In addition, introduction of the experimental data presented here into a mathematical model of electromechanical activity of the left ventricle or even of the whole heart might reveal the effects of *I*_Ks_ suppression on propensity of the heart to development of reentry-based arrhythmias and on ECG under control conditions and at adrenergic stimulation. In coexpression of WT and T309I subunits, a slight trafficking defect of WT subunits might contribute to the observed *I*_Ks_ channel dysfunction. Unfortunately, a direct proof of this suspicion is missing in the study.

### Conclusions

The c.926C > T-*KCNQ1* pore variant (p.T309I-Kv7.1) is the first LQTS-related founder mutation in Central Europe. Its dominant negative effect on *I*_Ks_ channel function is in contrast with the mild phenotype. As predicted by in silico modelling, the dysfunction may result in genesis of proarrhythmic DADs and action potential alternans at fast rates during β-adrenergic stimulation. Elimination of DADs by *I*_Ca_ modulation might be a possible new treatment strategy in some LQTS1 patients.

## Supplementary Information

Supplementary Information.

## Data Availability

The relevant data are available from the authors upon reasonable request.
