# Author Correction: Long-QT founder variant T309I-Kv7.1 with dominant negative pattern may predispose delayed afterdepolarizations under β-adrenergic stimulation

**DOI:** 10.1038/s41598-021-89234-z

**Published:** 2021-04-29

**Authors:** Iva Synková, Markéta Bébarová, Irena Andršová, Larisa Chmelikova, Olga Švecová, Jan Hošek, Michal Pásek, Pavel Vít, Iveta Valášková, Renata Gaillyová, Rostislav Navrátil, Tomáš Novotný

**Affiliations:** 1 Department of Medical Genetics, University Hospital Brno and Faculty of Medicine, Masaryk University, Jihlavská, 20, 625 00 Brno, Czech Republic; 2 Department of Experimental Biology, Faculty of Science, Masaryk University, Kotlářská 267/2, 611 37 Brno, Czech Republic; 3 Department of Physiology, Faculty of Medicine, Masaryk University, Kamenice 5, 625 00 Brno, Czech Republic; 4 Department of Internal Medicine and Cardiology, University Hospital Brno and Faculty of Medicine, Masaryk University, Jihlavská 20, 625 00 Brno, Czech Republic; 5 Department of Biomedical Engineering, Faculty of Electrical Engineering and Communication, Brno University of Technology, Technická 10, 616 00 Brno, Czech Republic; 6 Division of Biologically Active Complexes and Molecular Magnets, Regional Centre of Advanced Technologies and Materials, Faculty of Science, Palacký University in Olomouc, Šlechtitelů 27, 783 71 Olomouc, Czech Republic; 7 Institute of Thermomechanics, Czech Academy of Sciences, Dolejškova 5, 182 00 Prague, Czech Republic; 8 Department of Paediatrics, Faculty of Medicine, University Hospital Brno, Masaryk University, Černopolní 9, 613 00 Brno, Czech Republic; 9 Repromeda, Clinic for Reproductive Medicine and Preimplantation Genetic Diagnosis, Biology Park, Studentská 812/6, 625 00 Brno, Czech Republic

Correction to: *Scientific Reports* 10.1038/s41598-021-81670-1, published online 11 February 2021

This Article contains an error in concentration unit in Fig. 5c and Supplementary Figure S11 where,

“milimole (mM)”.

should read:

“micromole (µM)”.

The correct Figure 5c and S11 appear below as Figures 1 and  2 respectively.

**Figure 1:**
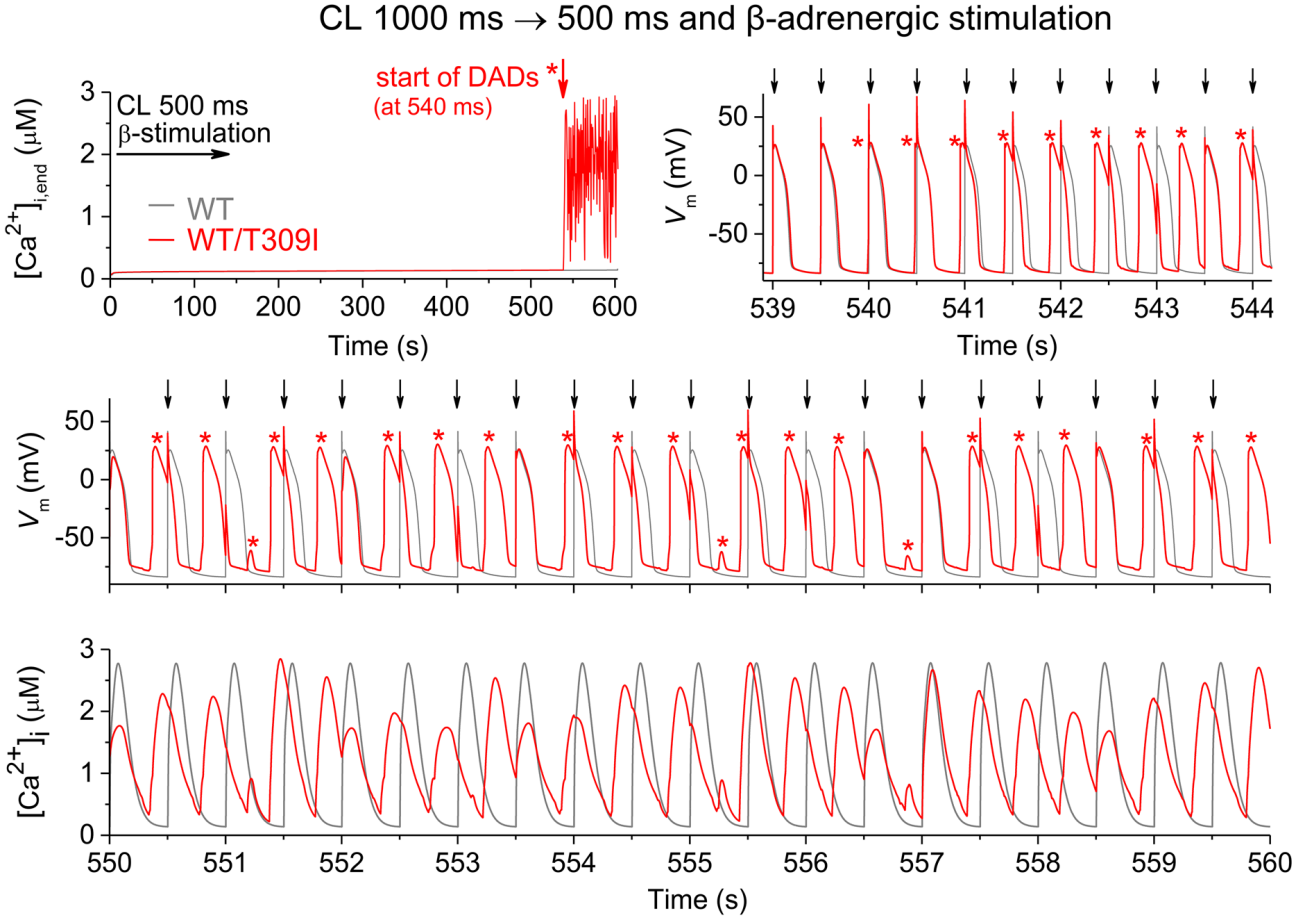
A correct version of the original Figure 5c.

**Figure 2:**
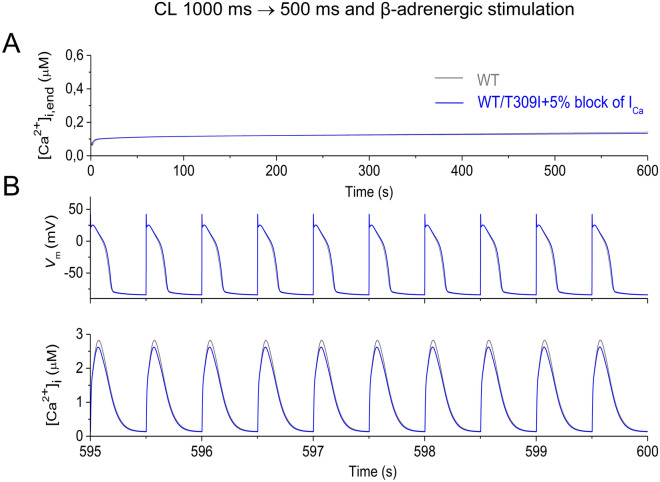
A correct version of the original Figure S11.

