# Supplementary material for: Long-QT founder variant T309I-Kv7.1 with dominant negative pattern may predispose delayed afterdepolarizations under β-adrenergic stimulation
Source: Sci Rep. 2021 Feb 11;11:3573. doi: 10.1038/s41598-021-81670-1 (PMC7878757; doi:10.1038/s41598-021-81670-1)
Supplement: Supplementary file 1 — Supplementary Information. [file 41598_2021_81670_MOESM1_ESM.pdf]

Long-QT founder variant T309I-Kv7.1 with dominant negative pattern may predispose delayed afterdepolarizations under β-adrenergic stimulation

SUPPLEMENTARY MATERIAL

Iva Synková, Markéta Bébarová, Irena Andršová, Larisa Chmelikova, Olga Švecová, Jan Hošek, Michal Pásek, Pavel Vít, Iveta Valášková, Renata Gaillyová, Rostislav Navrátil, Tomáš Novotný

Correspondence to:

Assoc. Prof. MUDr. Markéta Bébarová, Ph.D.

Department of Physiology
Faculty of Medicine
Masaryk University
Kamenice 5
625 00 Brno
Czech Republic

tel. +420-549493147, mbebar@med.muni.cz

Supplemental Methods

Clinical diagnostics

Patients with suspected LQTS are regularly investigated at the Department of Internal Medicine and Cardiology, and at the Department of Paediatrics (both at the University Hospital Brno and Faculty of Medicine, Masaryk University, Brno, Czech Republic). The diagnosis is established according to ESC Guidelines.[1] The study conformed to the principles outlined in the Declaration of Helsinki. All participants signed a written consent form prior to their inclusion in the study. In the case of participants under the age of 18 years, the written informed consent was obtained from a parent and/or legal guardian. The study was approved by the Multicenter Ethical Committee, University Hospital Brno (Brno, Czech Republic).

All individuals included in this study underwent clinical examination and bicycle ergometry to obtain ECG traces at different adrenergic states. A 12-lead ECG with Mason-Likar modification was used. The initial stress was set to 0.5 W/kg, and increased by 0.5 W/kg every three minutes to achieve a heart rate higher than the submaximal value with respect to age and sex.

All ECGs were recorded as paper printings at the speed of 50 mm/s and voltage of 20 mm/mV, and QT and RR intervals were measured manually for the periods of rest and in the fourth minute of the recovery period of the exercise test. In the majority of cases, the QT interval was measured in the lead V5; the other leads were used only when the end of the T wave could not be discriminated in this lead. In cases when the end of T wave was not clearly visible, the threshold method was used. The QT intervals were corrected for the respective heart rate using Bazett's formula: $QTc = QT/\sqrt{RR}$ (both intervals were measured in seconds).

Genetic testing

Between 2000 and 2018, 132 unrelated index cases with susceptibility to LQTS were

examined at the Department of Medical Genetics (University Hospital Brno and Faculty of Medicine, Masaryk University, Brno, Czech Republic). Informed consents and peripheral blood samples were collected from patients. DNA was extracted by standard molecular techniques. Molecular analysis of LQTS-associated genes including the *KCNQ1* gene was performed according to current practises for molecular genetics diagnostics. The classical method (multiplex PCR/SSCP analysis of 3 LQTS major genes) was followed by Sanger sequencing on ABI 3100 Genetic Analyser (*Applied Biosystems*™, Foster City, CA, USA). Primers for the screening of entire coding regions of genes *KCNQ1*, *KCNH2* and *SCN5A* were designed with Primer-BLAST tool.[2] Direct Sanger sequencing was then replaced with massive parallel sequencing (MPS) of 5 LQTS-related genes (*KCNQ1*, *KCNH2*, *SCN5A*, *KCNE1*, *KCNE2*) on GS Junior (*Roche*, Basel, Switzerland). The sequencing library in this case was prepared with a hybridization capture-based target enrichment method using NimbleGen SeqCap EZ Choice Library Kit (*Roche*, Basel, Switzerland). Since 2016, MPS of 13 LQTS-associated genes (*KCNQ1*, *KCNH2*, *SCN5A*, *AKAP9*, *CACNA1C*, *CALM1*, *CALM2*, *CAV3*, *KCNE1*, *KCNE2*, *KCNJ5*, *SCN4B*, *SNTA1*) has been performed on MiSeq (*Illumina*, San Diego, CA, USA). Two types of sequencing library have been used: 1) Amplicon library prepared with commercial kit TruSeq Custom Amplicon Kit (*Illumina*, San Diego, CA, USA); and 2) Target enriched library prepared with hybridization capture-based method using KAPA HyperPlus Kit with SeqCap EZ Choice Library Kit (*Roche*, Basel, Switzerland). The procedure has been performed as recommended by the manufacturer. Genetic counselling and testing of first-degree relatives have been offered to patients at risk.

In silico analysis

Various *in silico* tools were used to predict the possible clinical impact of the identified sequence variant (Suppl. Tab. S1). The functional impact of the amino acid substitution was

predicted with SIFT, Provean,[3] MutationTaster, FATHMM,[4] and PMUT.[5] The conservation of the impacted amino acid position was measured with LRT and MutationAssessor. The visualization of protein conservation across species was performed in MEGA7 software.[6] Protein sequences of included species were obtained from Ensembl Genome Browser (www.ensembl.org/index.html). Sequences were aligned with ClustalW with preset parameters. Allele frequency of the substitution was determined from online databases ExAC[7] and GnomAD.

Suppl. Tab. S1: *In silico* prediction of the *KCNQ1* substitution c.926C>T

Tool	Score	Result
SIFT	0	Damaging
Provean	-5.8	Damaging
MutationTaster	1	Disease-Causing
FATHMM	-5.03	Damaging
Pmut	0.94	Disease-Causing
LRT	0.00000199	Deleterious
MutationAssessor	4.1149	High

SIFT - The Sorting Intolerant From Tolerant algorithm, Provean - Protein variation effect analyzer, FATHMM - Functional Analysis through Hidden Markov Models (v2.3), LRT - Likelihood Ratio Test

Haplotype analysis

For the haplotype analysis, 9 STR (short tandem repeats) markers spanning the ~11.9-Mb region of chromosome 11 (including the *KCNQ1* gene) were chosen from UCSC Genome Browser: D11S1363, D11S922, D11S4046, D11S4088, D11S4146, D11S1760, D11S1338, D11S4149, D11S4116 (Suppl. Tab. S2). Multiplex PCR with fluorescently labelled primers and fragment analysis with capillary electrophoresis were performed on SeqStudio Genetic Analyzer (*Applied Biosystems*™, Foster City, CA, USA). The haplotype linked to the mutation was identified by studying segregation in families.

To obtain results with higher resolution and to identify possible crossing over spots, a SNP (single nucleotide polymorphism) marker analysis using HumanKaryomap-12 DNA

Analysis Kit (*Illumina*, San Diego, CA, USA) was also performed in the probands; 6219 SNPs on the p-arm of chromosome 11 were analysed.

Population allele frequency analysis was performed after identifying a common STR allele in the marker D11S4088 in all of the affected individuals. The control group was formed by 52 unrelated patients examined at the Department of Clinical Genetics, Faculty Hospital Brno, with a signed informed consent from each patient agreeing that their DNA samples could be used for clinical research. STR alleles (104) were amplified with fluorescently labelled primers and analysed with capillary electrophoresis.

Suppl. Tab. S2: Analysed STR markers and their distance to the mutation

marker	distance (bp)
D11S1363	-1542365
D11S922	-999468
D11S4046	-641034
c.926C>T	0
D11S4088	150263
D11S1923	633093
D11S4146	1137420
D11S1760	2779668
D11S1338	3383243
D11S4149	6525241
D11S4116	10345930
D11S902	14883773

(UCSC Genome Browser; www.genome.ucsc.edu/)

Biophysical analysis

Wild-type (WT) human *KCNQ1* in a pIRES2-eGFP vector and WT human *KCNE1* in a pKB-CMV vector were kindly provided by Prof. Paul G.A. Volders, MD, PhD (Maastricht University, Maastricht, Netherlands). *Yotiao* in a pGW1 vector was the kind gift of Prof. Robert R. Kass (Columbia University, New York, USA).

Plasmid DNA was cloned into chemically competent *Escherichia coli* cells from

NovaBlue Singles™ Competent Cells (*Novagen*, Madison, WI, USA) by the heat-shock technique according to the manufacturer's manual. After cultivation, plasmids were isolated from bacterial cells using endotoxin-free QIAprep Spin Miniprep Kit (*Qiagen*, Hilden, Germany). The quantity and purity of isolated plasmids were measured spectrophotometrically by BioPhotometer (*Eppendorf*, Hamburg, Germany).

The mutation c.926C>T in the human *KCNQ1* (p.T309I) was generated by site-directed mutagenesis using QuikChange II XL Site-Directed Mutagenesis Kit (*Agilent Technologies*, Cedar Creek, TX, USA) with the following primers:

5'-TGGTGGTGACTATGACCACCCCCCACC-3'

5'-GGTGGGGGGTGGTCATAGTCACCACCA-3'

The presence of the mutation in *KCNQ1* was verified by sequencing performed by the *Generi Biotech* company (Hradec Králové, Czech Republic).

TransFast Transfection Reagent (*Promega*, Madison, WI, USA) was used for transfection of the plasmids (*KCNQ1*, *KCNE1* and *Yotiao* in the molar ratio 1:2:4, total amount of DNA was 1 μg and the ratio of DNA to transfection agent was 1:1.5) into Chinese hamster ovary (CHO) cells that were cultured at 37°C / 5% CO_2 in Ham's F-12 medium supplemented with 10% foetal calf serum and 0.005% gentamycin (*Sigma-Aldrich*, St. Louis, MO, USA). *KCNQ1* was transfected in one of three ways: 1) WT variant alone (WT); 2) T309I variant alone (T309I); 3) both the WT and T309I variants cotransfected in the ratio 1:1 (WT/T309I) to mimic the heterozygous state in the mutation carriers.

Measurements were performed ~24 h after the transfection by the whole cell patch clamp technique in the voltage clamp mode at 37℃. The patch pipettes were pulled from borosilicate glass capillary tubes and heat-polished on a programmable horizontal puller (*Zeitz-Instruments Vertriebs GmbH*, Martinsried, Germany). The resistance of the filled glass electrodes was below 2.5 MΩ to keep the access resistance as low as possible. For the

generation of experimental protocols and data acquisition, the Axopatch 200A equipment and pCLAMP 9.2 software (*Molecular Devices,* San José, CA, USA) were used. The series resistance was compensated up to 60%. The measured ionic currents were digitally sampled at 2 kHz (after low-pass filtering at 5 kHz) and stored on the hard disc. For the experimental protocols, please see Results; if not mentioned, the stimulation frequency was 0.08 Hz. The average cell membrane capacitance was comparable in WT, T309I, and WT/T309I I_{Ks} channels (13.8 ± 1.7, 13.3 ± 2.0, and 13.5 ± 1.8 pF, respectively; $P > 0.05$).

Tyrode solution of the following composition was used to perfuse the measured cells (in mmol/L): NaCl 132, KCl 4.8, $CaCl_2$ 2.0, $MgCl_2$ 1.2, HEPES 10, glucose 5 (pH was adjusted to 7.4 with NaOH). The patch electrode filling solution contained (in mmol/L): K-aspartate 110, K_2ATP 5, $CaCl_2$ 1, $MgCl_2$ 1, EGTA 11, HEPES 10 (pH 7.3 adjusted with KOH). The junction potential was +15 mV. To simulate β-adrenergic stimulation, the pipette solution was supplemented with cyclic adenosine monophosphate (cAMP, 200 μmol/L) and inhibitor of serine/threonine phosphatases okadaic acid (OA, 0.2 μmol/L) in some experiments. The stock solutions were kept frozen and the chemicals were added to the pipette solution before the measurements were started. The pipette solution was subsequently kept in the fridge, lying on ice, before being filled to the measuring micropipette. The chemicals were purchased from *Sigma-Aldrich* (Prague, Czech Republic) unless otherwise indicated.

The voltage dependence of steady-state activation was fitted using the Boltzmann equation: $y = I_{max} / (1 + \exp((V_{1/2} - V) / k))$ to determine the half-maximal activation voltage $V_{1/2}$ and the slope factor k. Time courses of activation and deactivation were fitted with a single exponential function: $y = A \cdot (1 - \exp(-t/\tau))$, where A is amplitude and τ is the time constant.

Confocal microscopy

WT human *KCNQ1* tagged with GFP at the 3'-terminus or without GFP in a pBK-CMV vector (vector without GFP marker), as well as WT human *KCNE1* in a pBK-CMV vector were kindly provided by Prof. Paul G.A. Volders, MD, PhD (Maastricht University, Maastricht, Netherlands). The mutation c.926C>T (p.T309I) in the human *KCNQ1* with tagged GFP was generated by site-directed mutagenesis technique, as described above.

TransFast Transfection Reagent (*Promega*, Madison, WI, USA) was used for transfection of the plasmids (*KCNQ1* and *KCNE1* in the molar ratio 1:2, the total amount of DNA was 1 µg and the ratio of DNA to transfection agent was 1:1.5) into CHO cells. The cells were cultured at 37°C / 5% CO_2 in Ham's F-12 medium supplemented with 10% foetal calf serum and 0.005% gentamycin (*Sigma-Aldrich*, St. Louis, MO, USA) in glass bottom dishes (*Cellvis*, Mountain View, CA, USA) coated with fibronectin (*Sigma-Aldrich*, St. Louis, MO, USA) to enhance cell adhesion. After ~24 h, the CHO cells were transiently transfected in one of three ways: 1) WT variant alone (WT-GFP); 2) T309I variant alone (T309I-GFP); 3) both the WT and T309I variants cotransfected in the ratio 1:1 (WT-GFP/T309I-GFP). Additional experiments with cotransfected T309I-GFP and WT with no GFP were also performed to analyse cell membrane expression of the mutated subunits if cotransfected with WT. In these experiments, the cell membrane staining with CellMask™ Orange Plasma Membrane Stain (*Thermo Fisher Scientific*, Waltham, MA, USA) was used to identify distribution of GFP signal at the cell membrane.

Measurements were performed ~48 h after the transfection. A confocal laser scanning microscope Leica TCS SP8 X (*Leica microsystems*, Wetzlar, Germany) was used to analyse the subcellular localization of WT, T309I, and WT/T309I I_{Ks} channels. Excitation wavelength was set to 488 nm and emission range to 500-544 nm, corresponding to the GFP spectral properties. When the cells were labelled with CellMask™ Orange Plasma Membrane Stain,

the excitation wavelength was set to 554 nm and the detection range was set between 565 and 610 nm. The samples were observed using an oil lens 63X objective. The acquired images (at a spatial resolution of 1024 × 1024 pixels, physical length of 184.52 × 184.52 µm, and bit depth of 8 bit) were averaged three times and exported in the TIFF image format. GFP localization was analysed by relative intensity of line plot profiles.

Mathematical modelling

A previously published model of human ventricular myocyte[8] was modified and used for mathematical simulations. The principal modification includes: (i) incorporation of separate dyadic spaces adjacent to the tubular and surface membranes; (ii) incorporation of separate junctional sarcoplasmic reticulum (JSR) compartments adjacent to the t-tubular and surface dyadic spaces (JSR_t and JSR_s); (iii) incorporation of separate sub-sarcolemmal spaces adjacent to the t-tubular and surface membrane; and (iv) reformulation of description of I_{Ks} and I_{K1} to be more consistent with our new experimental data and data from literature.

The schematic diagram of the model is illustrated in Suppl. Fig. S1. The volume of the model cell (V_{tot}) and total volumes of myoplasm, network sarcoplasmic reticulum (NSR), junctional sarcoplasmic reticulum (JSR), and dyadic space (20.9, 14.2, 1.15, 0.088, and 0.00067 pL, respectively) were left the same as in our previous model.[8] The total volume of subsarcolemmal space (0.412 pL) was set to represent 2% of V_{tot};[9] the fractional volumes of surface and t-tubular subsarcolemmal spaces ($f_{Vs,s}$ = 0.49 and $f_{Vs,t}$ = 0.51, respectively) were set to be proportional to the non-junctional area of each membrane in the model cell (95.4% of the surface membrane and 79.4% of the t-tubular membrane).[10] The fractional volumes of t-tubular and surface dyadic spaces and of corresponding JSR compartments (0.8 and 0.2, respectively) were set to be proportional to the junctional area of each membrane in the model

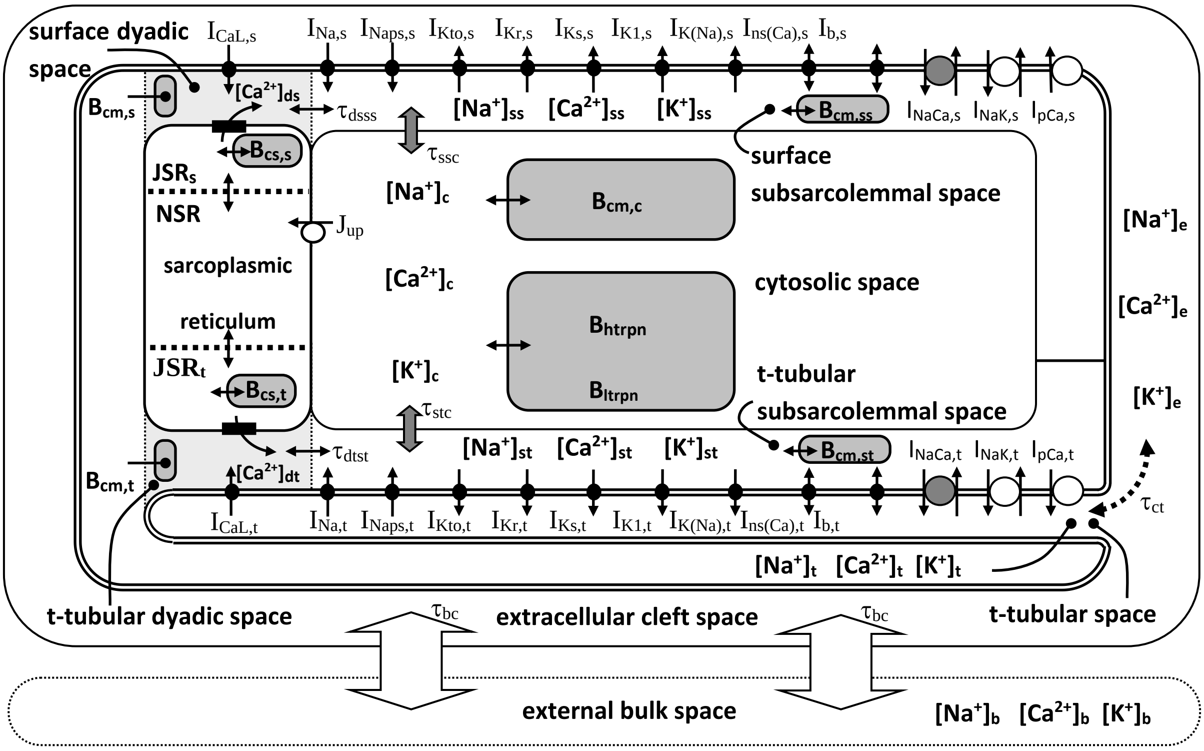

Suppl. Fig. S1: Schematic diagram of the modified model of human ventricular cell. Description of electrical activity of the surface (s) and t-tubular (t) membranes comprises formulations of the following ion currents: fast sodium current (I_{Na}), persistent sodium current (I_{Naps}), L-type calcium current (I_{CaL}), transient outward potassium current (I_{Kto}), rapid and slow components of delayed rectifier potassium current (I_{Kr} and I_{Ks}), inward rectifier potassium current (I_{K1}), background currents (I_b), sodium-activated potassium current ($I_{K(Na)}$), calcium-activated non-specific current ($I_{ns(Ca)}$), sodium-calcium exchange current (I_{NaCa}), sodium-potassium pump current (I_{NaK}), and calcium pump current (I_{pCa}). The intracellular space contains the cytosolic space (c), surface and t-tubular subsarcolemmal subspaces (ss, st), surface and t-tubular dyadic spaces (dt, ds), and network and junctional compartments of sarcoplasmic reticulum (NSR, JSR_s, JSR_t; for explanations of the abbreviations, see the text). J_{up} represents Ca^{2+} flow via SERCA and the small filled rectangles in JSR membrane ryanodine receptors. The small black and grey bi-directional arrows denote intracellular ion diffusion; the related ion fluxes between dyadic spaces, subsarcolemal spaces and cytosol are controlled by the time constants τ_{dsss}, τ_{dtst}, τ_{ssc}, and τ_{stc}. Ion diffusion between the t-tubular and cleft spaces (controlled by τ_{ct}) is represented by the dashed arrow, and between the cleft and external bulk spaces (controlled by τ_{bc}) by the thick white arrows.

cell. This implicates that the fraction of L-type Ca^{2+} channels in the t-tubular membrane $f_{CaL,t}$ and related $f_{KL,t}$ should be around 0.8.

The time constants related to the rate of ion diffusion from the dyadic spaces to the subsarcolemmal spaces (τ_{dsss} = 0.819 ms, τ_{dtst} = 0.214 ms) were set to be consistent with the rate of ion diffusion from dyads in our previous model.[8] The time constants of ion diffusion

from the subsarcolemmal spaces to the cytosol (τ_{ssc} = 2.8 ms, τ_{stc} = 2.7 ms) were adjusted to reflect the ratio of diffusional areas between both spaces and cytosol and to ensure the physiological magnitude of Ca^{2+} transients under the sarcolemma and in the bulk cytosol.[9]

To meet the experimental data showing that non-NCX Ca^{2+} transport (predominately mediated by the sarcolemmal Ca^{2+} pump, PMCA) is responsible for about 23% of total sarcolemmal Ca^{2+} extrusion in human induced pluripotent stem cells,[11] that mean AP duration at 90% repolarisation (APD_{90}) at the 15th stimulation pulse applied at 1 Hz lies within 271 ±13 ms,[12] and that inhibition of I_{K1} and I_{Ks} causes only a minor change of the human AP (prolongation of APD_{90} by 3 to 5%)[13], a partial modification of the parameters describing membrane transport system in ref.[8] was necessary. This included: (i) reduction of P_{Ca} and $I_{pCa,max}$ to 0.00173 cm/s and 0.5 μA/cm^2; (ii) substitution of the original description of I_{K1}[8] by the formulation proposed in ref.[14]; and (iii) change of conductivities related to I_{K1} (g_{K1}), I_{Kr} (g_{Kr}), and I_{Ks} (g_{Ks}) to 0.682, 0.276, and 0.014 mS/cm^2, respectively. Following these changes a readjustment of $I_{NaK,max}$, g_{Nab}, and g_{Cab} to 1.17 μA/cm^2, 0.564 μS/cm^2, and 1.1 μS/cm^2, respectively, was also needed to preserve the physiological levels of cytosolic ion concentrations of the model cell at rest ($[Ca^{2+}]_{c,rest}$ = 34 nM, $[Na^+]_{c,rest}$ = 7.4 mM, and $[K^+]_{c,rest}$ = 140 mM). The conductivities (g_x), permeabilities (P_x), or maximum current densities ($I_{x,max}$) of all ion transporters and their t-tubular fractions ($f_{x,t}$) are specified in Suppl. Tab. S3.

Suppl. Tab. S3: Electrical properties of ion transporters in the modified model of human ventricular cardiomyocyte.

g_{Na}	25 mS/cm^2	$f_{Na,t}$	0.57	$g_{K(Na)}$	0.129 mS/cm^2	$f_{K(Na),t}$	0.56
g_{Naps}	0.01 mS/cm^2	$f_{Naps,t}$	0.56	g_{Nab}	0.564 μS/cm^2*	$f_{Nab,t}$	0.56
P_{CaL}	0.00173 cm/s*	$f_{CaL,t}$	0.80*	g_{Cab}	1.1 μS/cm^2*	$f_{Cab,t}$	0.56
P_{KL}	0.0000032 cm/s	$f_{KL,t}$	0.80*	$P_{ns(Ca)}$	1.75 nm/s	$f_{ns(Ca),t}$	0.56
g_{Kto}	0.132 mS/cm^2	$f_{Kto,t}$	0.56	k_{NaCa}	0.15 nA/cm^2 · mM^4	$f_{NaCa,t}$	0.56
g_{Kr}	0.276 mS/cm^2*	$f_{Kr,t}$	0.56	$I_{NaK,max}$	1.17 μA/cm^2*	$f_{NaK,t}$	0.56
g_{Ks}	0.014 mS/cm^2*	$f_{Ks,t}$	0.56	$I_{pCa,max}$	0.5 μA/cm^2*	$f_{pCa,t}$	0.56*
g_{K1}	0.682 mS/cm^2*	$f_{K1,t}$	0.80				

The t-tubular fractions of ion transporters ($f_{x,t}$) were adopted from [8] except for $f_{Ca,t}$, $f_{KL,t}$, and $f_{pCa,t}$.*Modified values.

The basic behaviour of the model is illustrated in Suppl. Fig. S2 which shows the superimposed action potentials (APs), principal membrane currents, and ion concentrations in intracellular and extracellular compartments as recorded during 1, 2 and 3 Hz steady-state stimulation. Similarly, as our previous model[8] the modified model reproduces well the frequency-dependent shortening of AP and increase of cytosolic Ca^{2+} and Na^{+} concentrations observed in human ventricular myocytes[15,16]; the model reconstruction of rate dependent changes of AP configuration published by Li et al.[12] is illustrated in Suppl. Fig. S3. A critical point of the present simulations is a proper formulation of $g_{Ks.}$ As demonstrated by Suppl. Fig. S4, the effect of total suppression of I_{Ks} on AP in the presented model (increase of APD_{90} by 5.4%) is fully consistent with that observed in the previously published and validated model by O'Hara et al. [17] that is freely available in Matlab or CellML codes.

To simulate changes of the model behaviour under β-adrenergic stimulation, some transport and Ca^{2+} buffering mechanisms of the model cell were reformulated to reflect cellular effects of 1 µM isoproterenol described in the Supplement to ref.[18] The reformulation includes: (i) increase of g_{Na} to 32.5 mS/cm^2; (ii) increase of P_{Ca} to 0.00433 cm/s; (iii) increase of g_{Ks} to 0.0717 mS/cm^2; (iv) incorporation of description of I_{Kb} based on the Ohm low with conductivity of 0.0075 mS/cm^2 (density of I_{Kb} in the t-tubular and surface membranes is assumed to be the same); (v) increase of Ca^{2+} release rate from JSR_t and JSR_s to corresponding dyadic spaces 1.75 times; (vi) decrease of NSR Ca^{2+}-ATPase forward half-saturation constant to 90.7 nM; (vii) decrease of $[Na^+]_c$ half saturation constant of I_{NaK} to 7 mM; and (viii) decrease of Ca^{2+} on rate constants for high and low affinity sites of troponin to 12500 and 25000 mM^{-1} s^{-1}, respectively. A reconstruction of the effects of β-adrenergic stimulation on AP (increased amplitude and decreased AP duration) and on cytosolic Ca^{2+} transient (increased amplitude) at 3.3 Hz stimulation published in the Supplement to ref.[18] is illustrated in Suppl. Fig. S5.

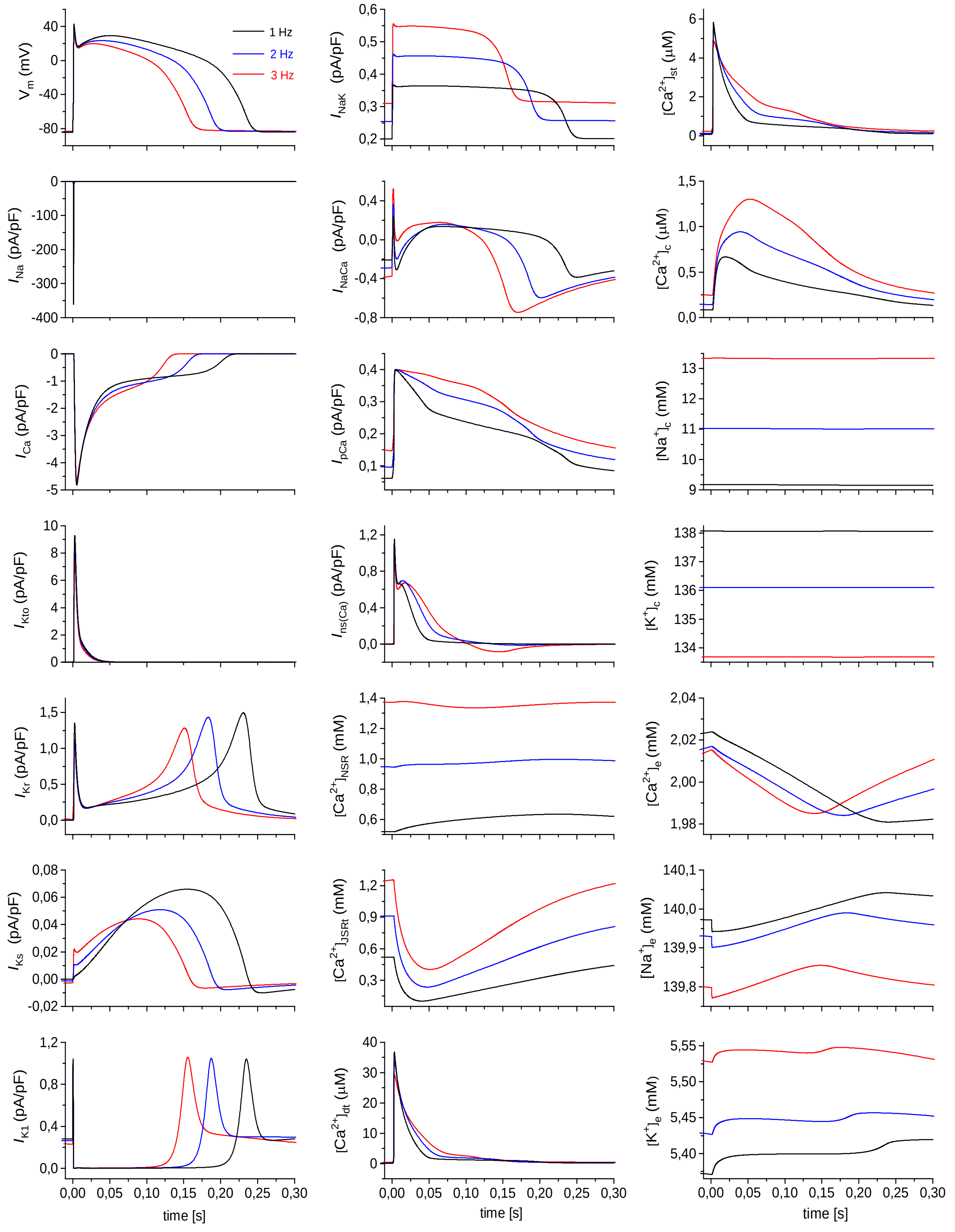

Suppl. Fig. S2: Membrane voltage (V_m), currents (I_{Na}, I_{Ca}, I_{Kto}, I_{Kr}, I_{Ks}, I_{K1}, I_{NaK}, I_{NaCa}, I_{pCa}, $I_{ns(Ca)}$), Ca^{2+} concentration changes in NSR ($[Ca^{2+}]_{NSR}$), JSR$_t$ ($[Ca^{2+}]_{JSRt}$), t-tubular dyadic space ($[Ca^{2+}]_{dt}$), t-tubular subsarcolemmal space ($[Ca^{2+}]_{st}$) and ion concentration changes in cytosolic space ($[Ca^{2+}]_c$, $[K^+]_c$, $[Na^+]_c$) and extracellular cleft space ($[Ca^{2+}]_e$, $[K^+]_e$, $[Na^+]_e$) during 1, 2 and 3 Hz steady-state stimulation in the model of human epicardial myocyte. All traces represent steady states after 600 s of real time stimulation under body temperature and external bulk ion concentrations: $[Na^+]_b$ = 140 mM, $[K^+]_b$ = 5.4 mM, $[Ca^{2+}]_b$ = 2 mM.

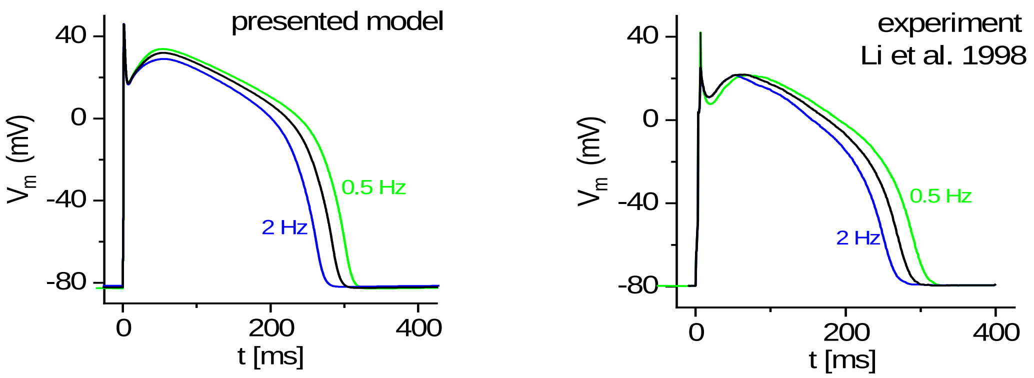

Suppl. Fig. S3: Comparison of model reconstruction of APs recorded at 0.5, 1 and 2 Hz with representative human subepicardial AP waveforms digitized from Li et al.[12]. To meet the experimental conditions reported by Li et al.[12], the extracellular cleft ion concentrations $[Na^+]_e$, $[K^+]_e$, and $[Ca^{2+}]_e$ were fixed at 136, 5.4 and 2 mM, respectively, and $[K^+]_i$ was fixed at 130 mM. Individual traces represent 15th AP from resting state.

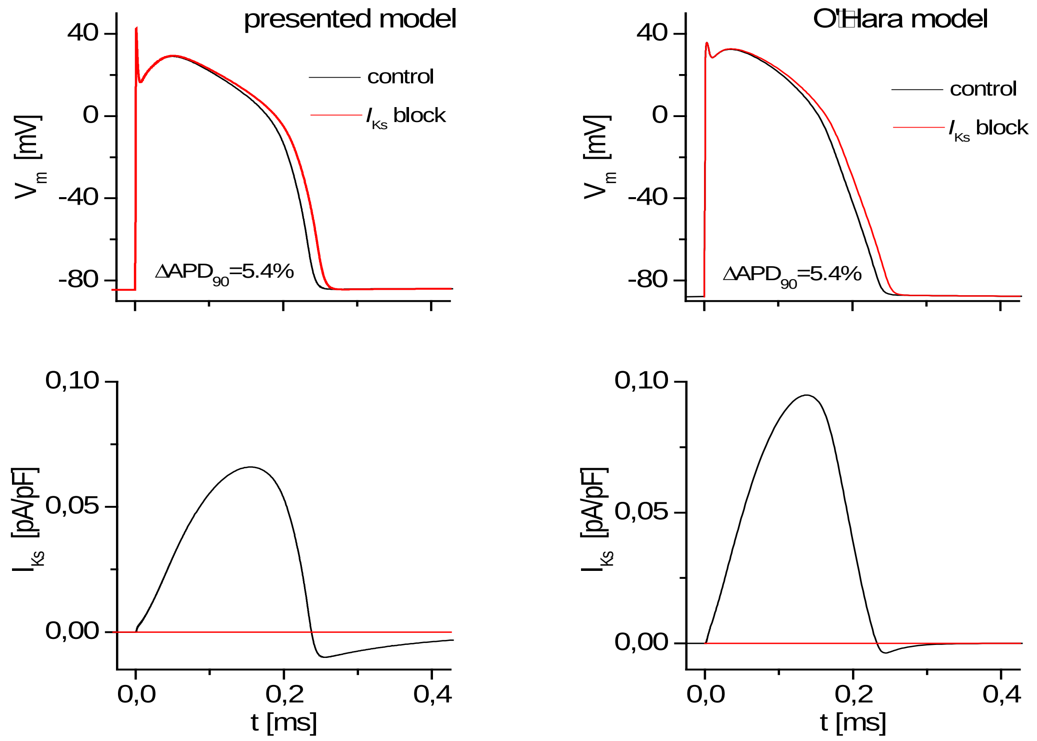

Suppl. Fig. S4: Comparison of the effect of total suppression of I_{Ks} on AP in the presented model and in the validated model of human epicardial myocyte published by O'Hara et al.[17]. The red traces represent the first AP elicited from 1 Hz steady state after I_{Ks} suppression.

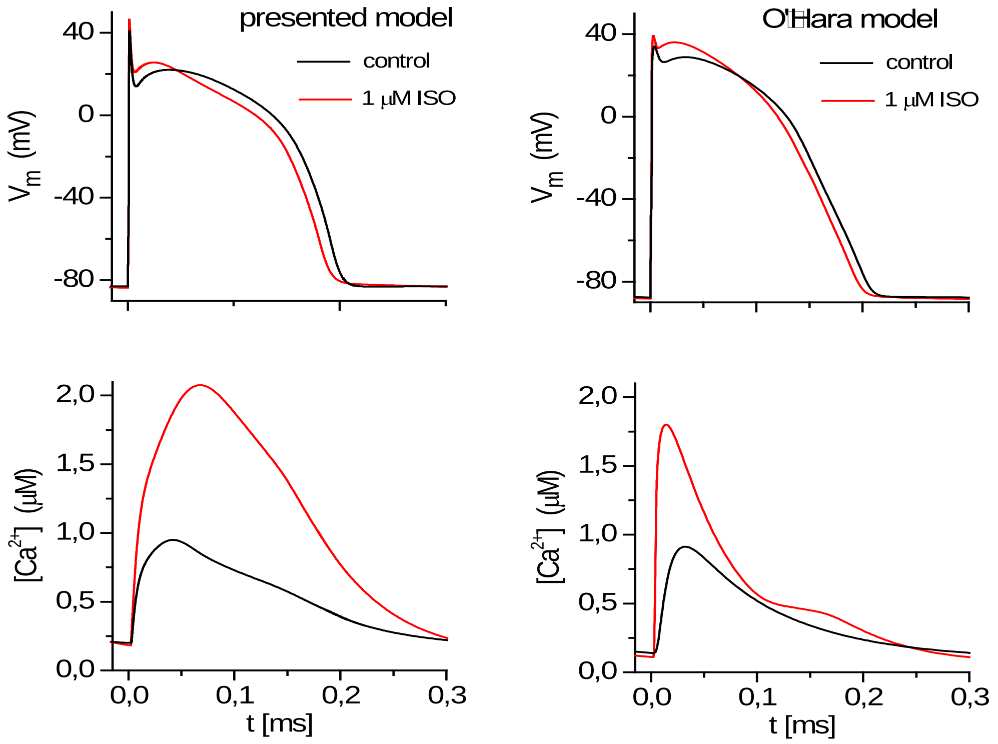

Suppl. Fig. S5: Model reconstruction of the effect of β-adrenergic stimulation on AP configuration and cytosolic Ca^{2+} transient $[Ca^{2+}]_c$ presented in the supplement to O'Hara et al.[17]. The traces represent simulated responses of both models after 30 s pacing at 3.3 Hz in control conditions (black lines) and under the effect of 1 μM isoproterenol (red lines). To meet the conditions used in O'Hara et al.[17] model, the extracellular cleft ion concentrations $[Na^+]_e$, $[K^+]_e$, and $[Ca^{2+}]_e$ were fixed at 140, 5.4 and 1.8 mM, respectively, in the presented model.

The gating properties of WT and WT/T309I I_{Ks} channels were incorporated into the model to comply with our experimental data (Suppl. Fig. S6). Beside this, an additional reduction of g_{Ks} to 40% of the control value was necessary to simulate the experimentally observed reduction of I_{Ks} caused by the WT/T309I mutation (Fig. 2B in the main text). To attain a dynamic steady state at all stimulation frequencies, the model was paced for 600 s of equivalent cell lifetime at external Na^+, K^+, and Ca^{2+} concentrations set to 140, 5.4, and 2 mmol/L, respectively. The numerical solution of the system of equations describing the model cell function was performed using the computational software MATLAB v. 7.2 (MathWorks, Inc.).

Statistical analysis

The data are mostly presented by the arithmetic mean (± SD from *n* patients, or ± SEM from *n* cells; Origin, version 8.5.1; *OriginLab Corporation*). To determine statistical significance of differences, paired/unpaired *t*-tests, and one-way/repeated measures ANOVA with the Bonferroni post-test were used; $P < 0.05$ was considered statistically significant. If the difference between the arithmetic and geometric means was >10%, the geometric mean x $^{x}/$ geometric SE (geometric SE = geometric SD$^{1/\sqrt{n}}$) and the non-parametric Mann-Whitney test were used (Figs. 2B and 3B in the main text file). To compare the statistical significance of differences in relative fluorescence intensity in Fig. 4 in the main text file (where several data samples did not show the normal distribution according to the Shapiro-Wilk test; the mean values are represented by the geometric mean ± 95% confidence interval), either the Friedman test or the Kruskal-Wallis test (both with the Dunn´s multiple comparison) were used, the first one in the case of paired data (comparison within individual groups), the latter in the case of unpaired data (comparison among various groups). The curve fitting and the statistical tests were performed using the GraphPad Prism, version 6.05 (*GraphPad Software, Inc.*).

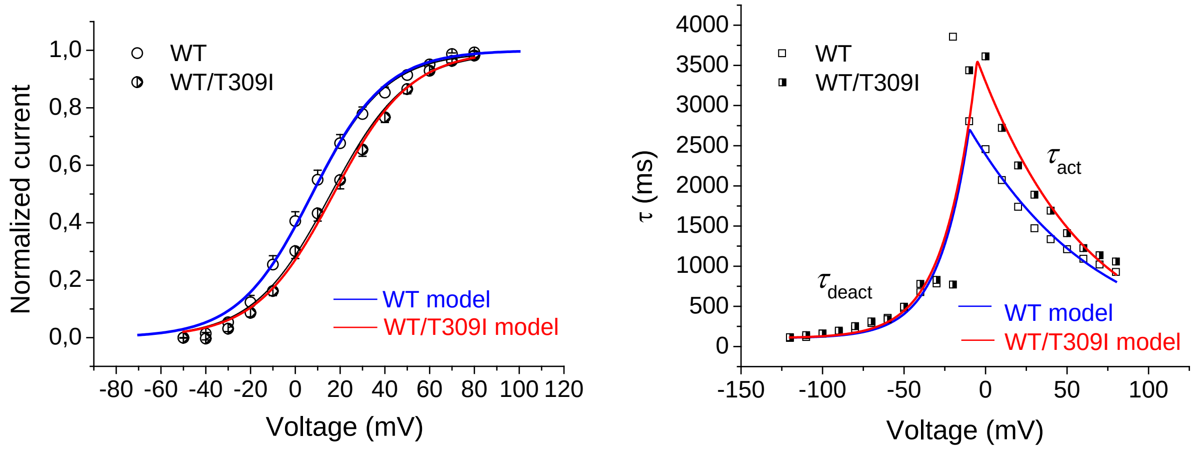

Suppl. Fig. S6: Voltage dependence of steady-state activation (A) and time constants of activation and deactivation (τ_{act} and τ_{deact}, respectively; B) in the model of human ventricular cell[8] after incorporation of the gating properties of WT (blue lines) and WT/T309I (red lines) I_{Ks} channels (experimental data are represented by empty and half-filled symbols, respectively). Lines in the part A were fitted using the Boltzmann equations Eq. 1 and Eq. 2 and lines in the part B were fitted using the Eq. 3 and Eq. 4 (below).

$$I_{\mathrm{WT}} = \frac{1}{1 + e^{(7.5 - \mathrm{V})/16.5}} \quad (1)$$

$$I_{\mathrm{WT/T309I}} = \frac{1}{1 + e^{(17.08 - \mathrm{V})/17.26}} \quad (2)$$

$$\tau_{\mathrm{WT}} = 0.105 + \frac{40}{\left(1 + e^{(-(\mathrm{V}+10)/0.2))^{0.01333}}\right) \cdot \left(1 + e^{((\mathrm{V}+90)/129))^{2.6}}\right)} \quad (3)$$

$$\tau_{\mathrm{WT/T309I}} = 0.105 + \frac{40}{\left(1 + e^{(-(\mathrm{V}+5.6)/0.2))^{0.01333}}\right) \cdot \left(1 + e^{((\mathrm{V}-90)/140))^{6}}\right)} \quad (4)$$

Supplemental Results

Clinical diagnostics

Representative examples of electrocardiograms (EGCs) of two T309I carriers, recorded at rest and in the 4th minute of recovery after the exercise, are shown in Suppl. Fig. S7, the first one recorded in a female who suffered cardiac arrest and was successfully resuscitated (parts A and B), the second one from an asymptomatic female (parts C and D). For results characterizing differences in QTc lengths in T309I carriers and their unaffected relatives, see Fig. 1A in the main text.

No significant changes were apparent in PQ interval and QRS complex (Suppl. Fig. S8). In the population over 16 years old, a non-significant tendency to a prolonged PQ interval (P = 0.07) and a shortened QRS complex (P = 0.11) was apparent in T309I carriers.

A

T309I, symptomatic female – at rest before start of the exercise test

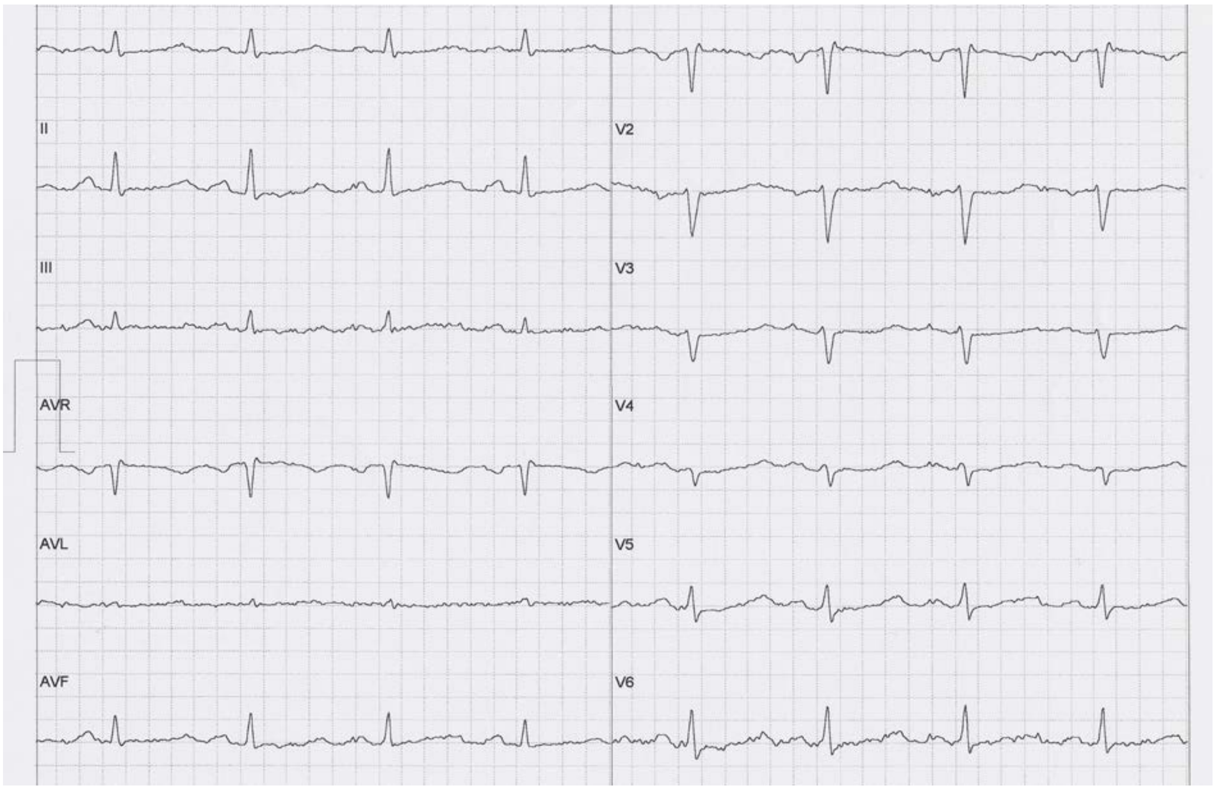

B

T309I, symptomatic female – in the 4th min of recovery after the exercise

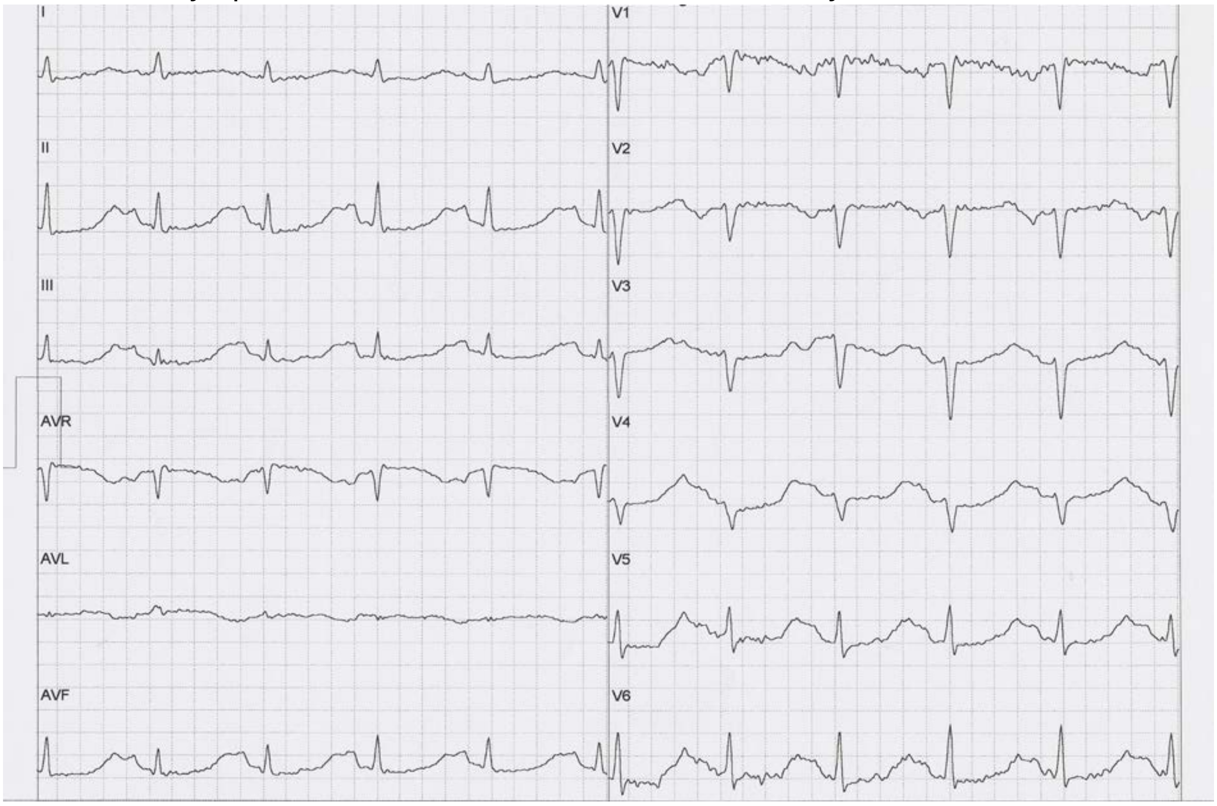

C

T309I, asymptomatic female – at rest before start of the exercise test

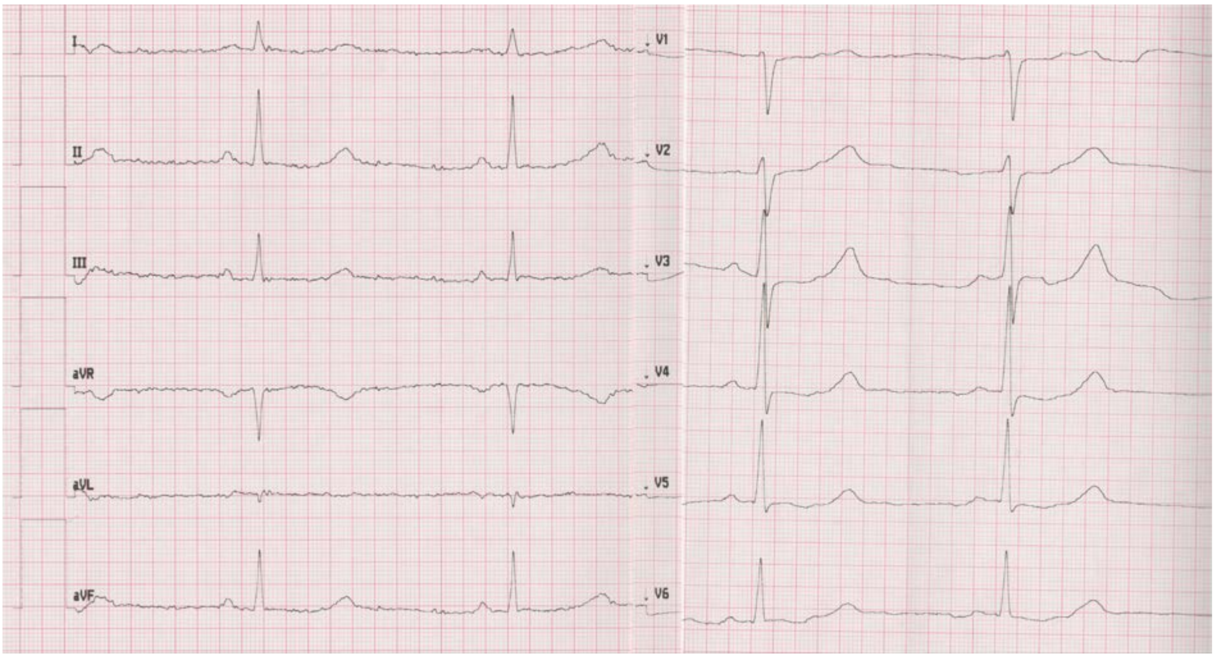

D

T309I, asymptomatic female – in the 4th min of recovery after the exercise

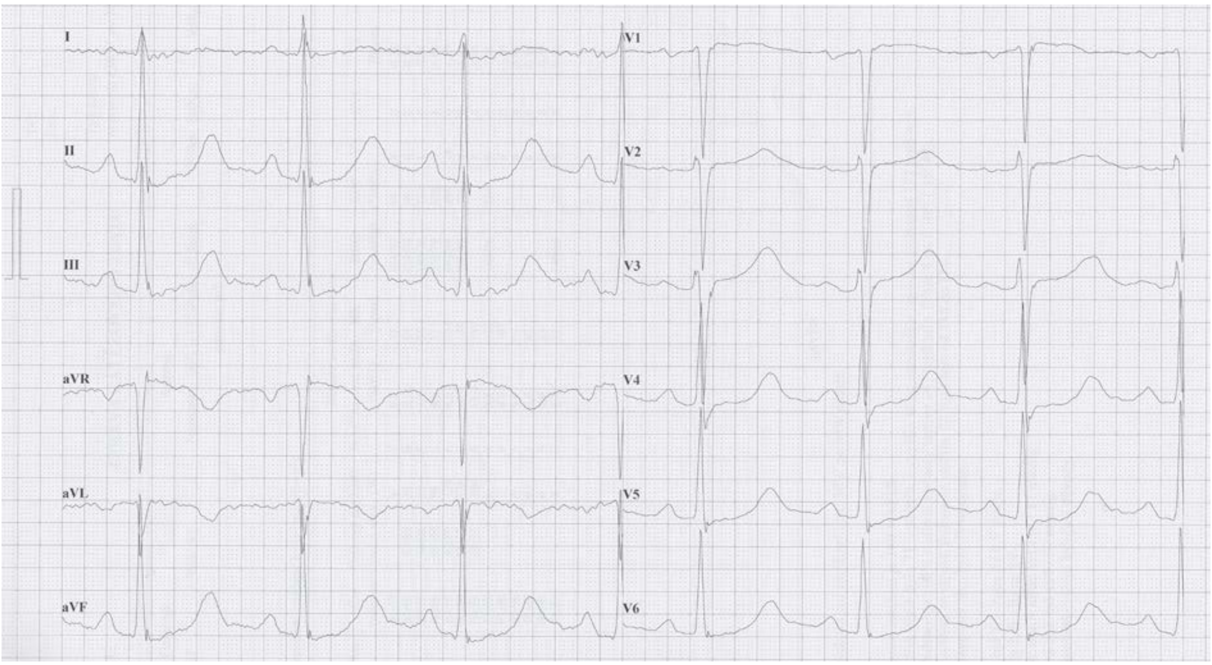

Suppl. Fig. S7: ECG examples of T309I carriers. A and B: ECG recordings (50 mm/s, 20 mm/mV) of an adult female with T309I variant who suffered cardiac arrest and was successfully resuscitated. The recordings were taken 6 months after the event during bicycle ergometry, before the exercise (sitting on the ergometer, A – QTc = 500 ms) and in the 4th minute of recovery after the exercise (B – QTc = 530 ms). C and D: ECG recordings (50 mm/s, 20 mm/mV) of an asymptomatic adult female with T309I variant at rest (C – QTc = 480 ms) and in the 4th minute of recovery after the exercise (D – QTc = 500 ms).

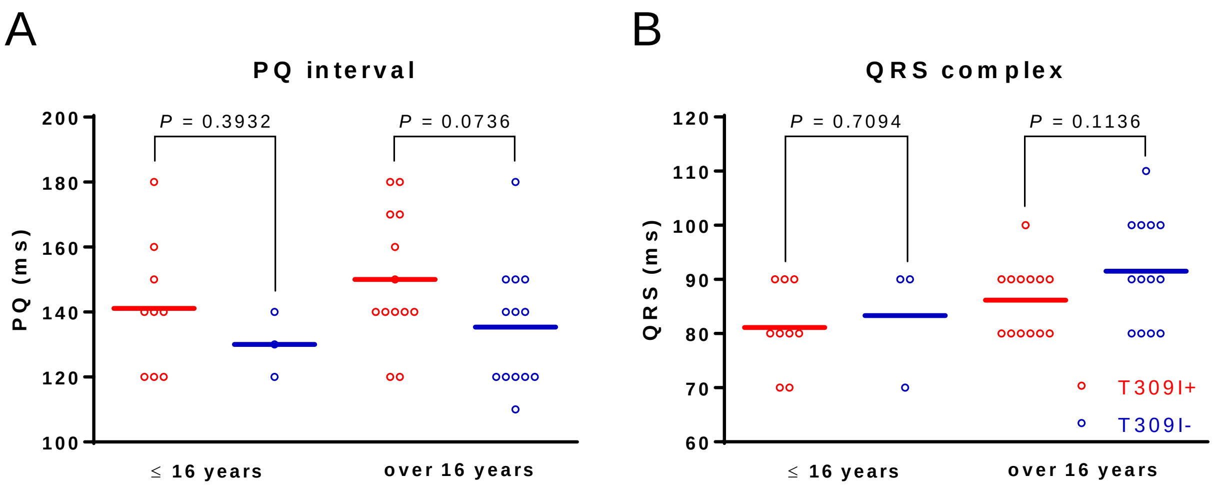

Suppl. Fig. S8: PQ interval and QRS complex durations in T309I carriers (T309I+, red circles) and in their unaffected relatives (T309I-, blue circles) as observed in persons up to 16 years old and in persons over 16 years old. No significant changes were observed between T309I+ and T309I-.

Genetic testing

In our cohort of 132 patients, pathogenic or probably pathogenic variants in any of LQTS-related genes were identified in 74 probands, mostly in the *KCNQ1* gene (in 44 probands, ~59%). The same substitution c.926C>T (NM_000218.2) in the *KCNQ1* gene (Suppl. Fig. S9) resulting in T309I amino acid change in the P-loop of Kv7.1 protein was identified in 10 unrelated probands out of all the 44 probands (~23%). Presence of other pathogenic variants (including other LQTS-related genes) was negated in all index cases.

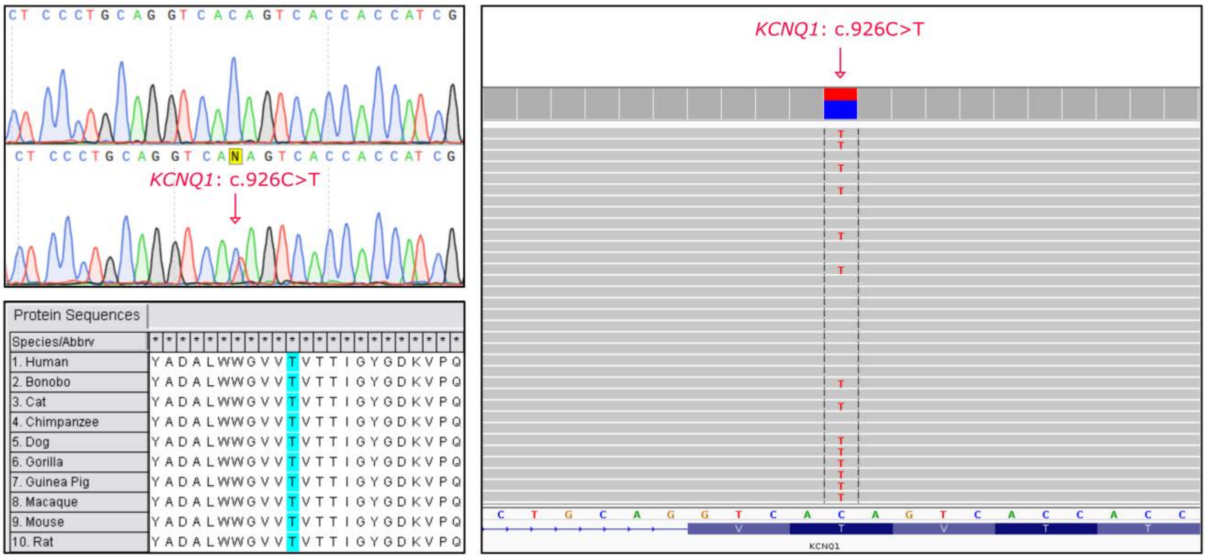

Suppl. Fig. S9: Genetic characterization of c.926C>T-*KCNQ1* variant. Results of sequencing analysis and evolutionary conservation analysis; left upper panel: variant c.926C>T detected by the Sanger sequencing, right panel: result of massive parallel sequencing, left lower panel: analysis of region 291-327 of the Kv7.1 human protein - threonine at the position 309 (where the T309I variant is located - highlighted in blue) is highly conserved in the analysed species.

Mathematical modelling

A detailed view on the mechanisms of development of delayed afterdepolarisations (DADs) observed in the model with WT/T309I mutation under β-adrenergic stimulation provides Suppl. Fig. S10. As indicated by asterisks in the figure, DADs were initiated by premature realise of Ca^{2+} from JSR (due to the SR Ca^{2+} overload at the time of 540 s) and consequent steep increase of inward components of I_{NaCa} and $I_{ns(Ca)}$.

The proarrhythmic action of WT/T309I channels was prevented by 5%-inhibition of cardiac calcium current (I_{Ca}); contemporary change in the peak value of Ca^{2+} transient was negligible (Suppl. Fig. 11).

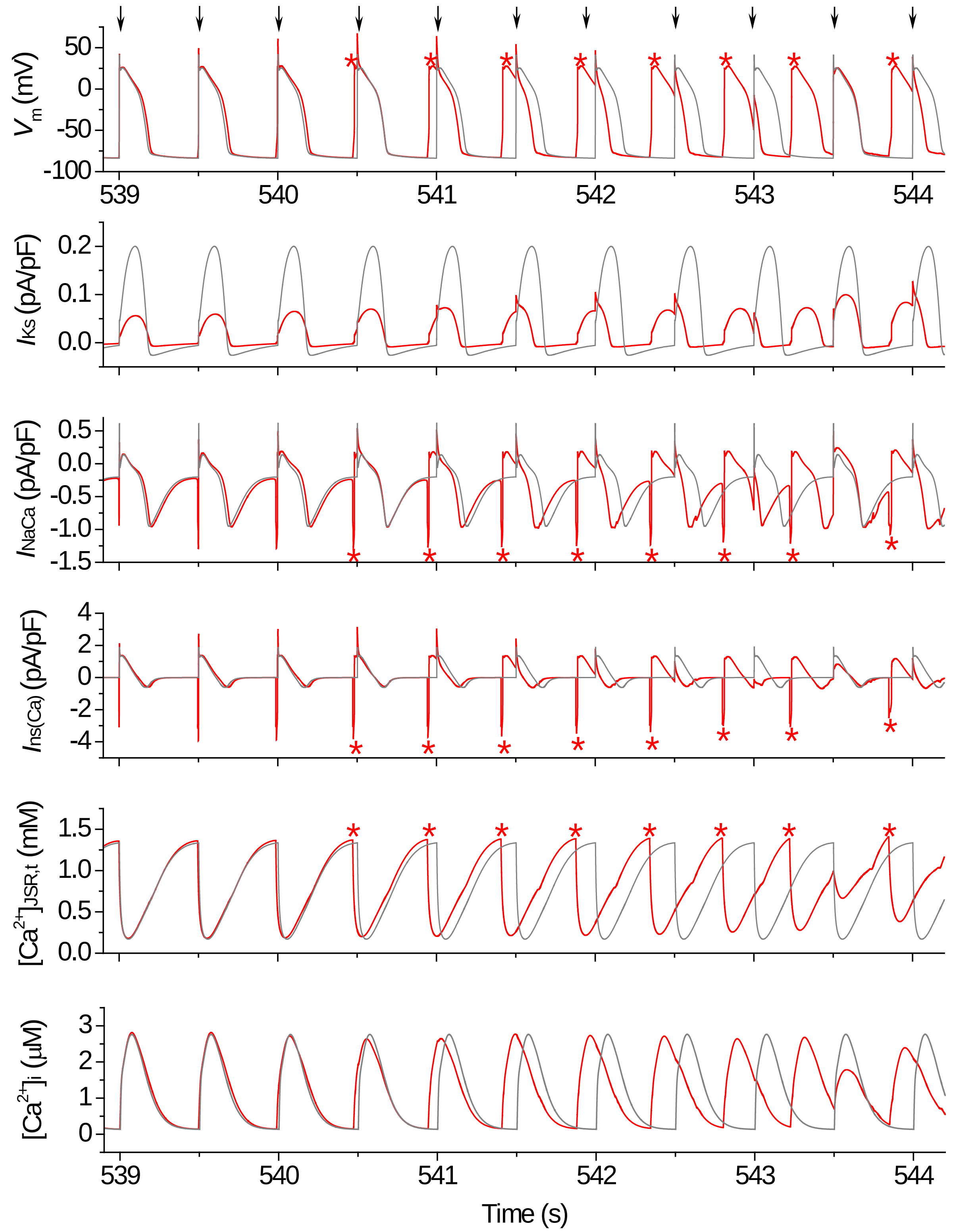

Suppl. Fig. S10: Onset of DADs in the model with WT/T309I dysfunctional channels under cycle length (CL) 500 ms and β-adrenergic stimulation. The individual graphs from up to down represent action potentials, membrane currents I_{Ks}, I_{NaCa} and $I_{ns(Ca)}$, changes of Ca^{2+} concentration in JSR_t, and cytosolic Ca^{2+} transients in the control model (grey line) and in the model with WT/T309I mutation (red line) 539 s after β-adrenergic stimulation and 2 Hz stimulation. The asterisks show the premature release of Ca^{2+} from JSR_t due to SR Ca^{2+} overload and subsequent activation of Ca^{2+} dependent currents (I_{NaCa} and $I_{ns(Ca)}$) responsible for DAD.

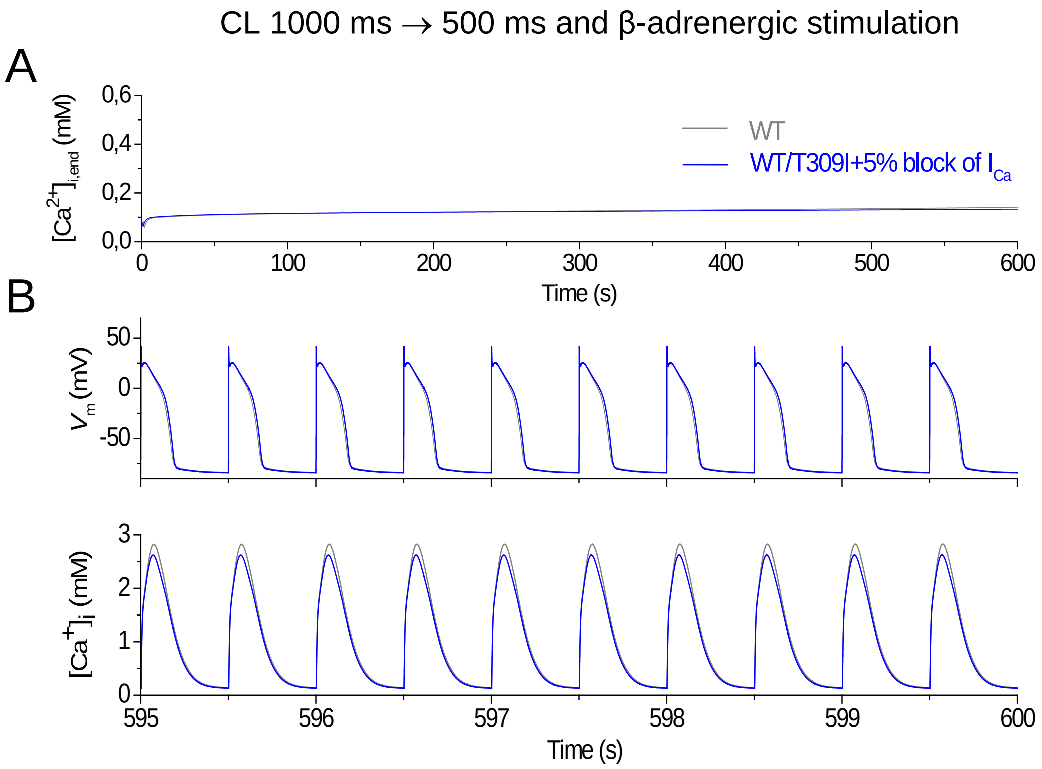

Suppl. Fig. S11: Proarrhythmic action of WT/T309I channels under β-adrenergic stimulation at the cycle length (CL) of 500 ms may be prevented by a slight inhibition of cardiac calcium current (I_{Ca}). If I_{Ca} was inhibited by 5% in WT/T309I model, the intracellular Ca^{2+} load (reflected by the level of $[Ca^{2+}]_{i,end}$) was comparable to that in WT model without I_{Ca} inhibition (A) and DADs were not generated (B; compare with Fig. 5C in the main text file). The action potential prolongation in WT/T309I model was negligible under these conditions (APD_{90} longer by 3% in WT/T309I model than in WT model; B, upper panel). The peak value of Ca^{2+} transient was diminished by 7% (B, lower panel).

References:

1. Priori SG, Blomström-Lundqvist C, Mazzanti A, et al. 2015 ESC Guidelines for the management of patients with ventricular arrhythmias and the prevention of sudden cardiac death: The Task Force for the Management of Patients with Ventricular Arrhythmias and the Prevention of Sudden Cardiac Death of the European Society of Cardiology (ESC). Endorsed by: Association for European Paediatric and Congenital Cardiology (AEPC). Eur Heart J. 2015;36(41):2793-2867.

2. Ye J, Coulouris G, Zaretskaya I, Cutcutache I, Rozen S, Madden TL. Primer-BLAST: a tool to design target-specific primers for polymerase chain reaction. BMC Bioinformatics. 2012;13:134.

3. Choi Y, Chan AP. PROVEAN web server: a tool to predict the functional effect of amino acid substitutions and indels. Bioinformatics. 2015;31(16):2745-2747.

4. Shihab HA, Gough J, Mort M, Cooper DN, Day INM, Gaunt TR. Ranking non-synonymous single nucleotide polymorphisms based on disease concepts. Hum Genomics. 2014;8:11.

5. López-Ferrando V, Gazzo A, de la Cruz X, Orozco M, Gelpí JL. PMut: a web-based tool for the annotation of pathological variants on proteins, 2017 update. Nucleic Acids Res. 2017;45(Web Server issue):W222-W228.

6. Kumar S, Stecher G, Tamura K. MEGA7: Molecular Evolutionary Genetics Analysis Version 7.0 for Bigger Datasets. Mol Biol Evol. 2016;33(7):1870-1874.

7. Lek M, Karczewski KJ, Minikel EV, et al. Analysis of protein-coding genetic variation in 60,706 humans. Nature. 2016;536(7616):285-291.

8. Hrabcová D, Pásek M, Šimurda J, Christé G. Effect of ion concentration changes in the limited extracellular spaces on sarcolemmal ion transport and Ca^{2+} turnover in a model of human ventricular cardiomyocyte. Int J Mol Sci. 2013;14(12):24271-24292.

9. Shannon TR, Wang F, Puglisi J, Weber C, Bers DM. A Mathematical Treatment of Integrated Ca Dynamics within the Ventricular Myocyte. Biophys J. 2004;87(5):3351-3371.

10. Page E, Surdyk-Droske M. Distribution, surface density, and membrane area of diadic junctional contacts between plasma membrane and terminal cisterns in mammalian ventricle. Circ Res. 1979;45(2):260-267.

11. Hwang HS, Kryshtal DO, Feaster TK, et al. Comparable calcium handling of human iPSC-derived cardiomyocytes generated by multiple laboratories. J Mol Cell Cardiol. 2015;85:79-88.

12. Li G-R, Feng J, Yue L, Carrier M. Transmural heterogeneity of action potentials and I_{to1} in myocytes isolated from the human right ventricle. Am J Physiol-Heart Circ Physiol. 1998;275(2):H369-H377.

13. Jost N, Virág L, Comtois P, et al. Ionic mechanisms limiting cardiac repolarization reserve in humans compared to dogs: Weak I_{K1}, I_{Ks} limit human repolarization reserve. J Physiol. 2013;591(17):4189-4206.

14. Fink M, Noble D, Virag L, Varro A, Giles WR. Contributions of HERG K+ current to repolarization of the human ventricular action potential. Prog Biophys Mol Biol. 2008;96(1-3):357-376.

15. Schmidt U, Hajjar RJ, Helm PA, Kim CS, Doye AA, Gwathmey JK. Contribution of

Abnormal Sarcoplasmic Reticulum ATPase Activity to Systolic and Diastolic Dysfunction in Human Heart Failure. J Mol Cell Cardiol. 1998;30(10):1929-1937.

16. Pieske B, Maier LS, Piacentino V, Weisser J, Hasenfuss G, Houser S. Rate Dependence of $[Na^+]_i$ and Contractility in Nonfailing and Failing Human Myocardium. Circulation. 2002;106(4):447-453.

17. O'Hara T, Virág L, Varró A, Rudy Y. Simulation of the Undiseased Human Cardiac Ventricular Action Potential: Model Formulation and Experimental Validation. McCulloch AD, ed. PLoS Comput Biol. 2011;7(5):e1002061.

18. O'Hara T, Rudy Y. Arrhythmia formation in subclinical ("silent") long QT syndrome requires multiple insults: Quantitative mechanistic study using the KCNQ1 mutation Q357R as example. Heart Rhythm. 2012;9(2):275-282.
